# Metabolic Glycan Imaging by Isonitrile–Tetrazine Click Chemistry

**DOI:** 10.1002/cbic.201300130

**Published:** 2013-05-13

**Authors:** Shaun Stairs, André A Neves, Henning Stöckmann, Yelena A Wainman, Heather Ireland-Zecchini, Kevin M Brindle, Finian J Leeper

**Affiliations:** [a] Department of Chemistry, University of CambridgeLensfield Road, Cambridge, CB2 1EW (UK) E-mail: fjl1@cam.ac.uk; [b] Cancer Research UK Cambridge Research Institute, Li Ka Shing CentreRobinson Way, Cambridge, CB2 0RE (UK)

**Keywords:** bioorthogonal chemistry, click chemistry, fluorescent probes, glycans, isonitrile, tetrazines

Bioorthogonal chemistry is the specific and exclusive reaction between a probe and a reporter group in the presence of the varied functionality present in biological systems.^1^, ^2^ In the chemical-reporter strategy, a biologically inert functional group is incorporated into the desired biomolecule, and, at the appropriate time, the reaction with a complementary abiotic functionality linked to various probes allows specific labelling of the biomolecule.^3^ This strategy has been used to label various biomolecules including proteins, lipids and glycans.^4^–^7^

Glycans are complex biological macromolecules consisting of covalently linked scaffolds of monosaccharide units. Most glycans exist as membrane-bound glycoconjugates, but many are secreted and become part of the extracellular matrix. They have important roles in intercellular signalling, cell adhesion and motility, and have also been shown to play a pivotal role in cancer onset and progression.^8^ In many cancer phenotypes, certain sugars, particularly sialic acid, are overexpressed, thus glycan imaging has become an attractive target for clinical development.^9^, ^10^ We have recently demonstrated that glycan imaging can be used to detect tumours in vivo.^11^

Labelling of glycoproteins in living systems presents a particular challenge as the chemical reporter must be introduced metabolically. This is achieved by feeding to the cells a sugar which is a metabolic precursor with an additional chemical reporter group attached. This sugar is then incorporated by the intracellular machinery into the growing glycan. The size and charge of the chemical reporter are of key importance if the precursor is to be accepted by the host cell's enzymatic pathways. Metabolic glycan labelling has currently only been achieved by using azide,^9^, ^12^–^14^ alkyne,^15^ ketone,^16^ thiol,^17^ alkene^18^ and cyclopropene^19^–^21^ groups as the chemical reporter group. The alkyne chemical reporter requires a copper catalyst for ligation with an azide, thus its utility for live-cell experiments is limited, although biocompatible copper catalysts are being developed.^22^ Ketone group labelling is achieved by reaction with a hydrazide probe; this presents two major problems: the reaction is not truly bioorthogonal, as ketones are found in many biomolecules, and reduced pH values are required for efficient ligation. Thiosugars can be labelled with electrophiles such as maleimides, but the thiol group is also not truly bioorthogonal and is present on many biomolecules. An unactivated alkene reacts very slowly with tetrazines,^18^ but a cyclopropene group reacts considerably faster and has recently been successfully incorporated into cell-surface glycans, initially by using a cyclopropene-conjugated sialic acid^19^ but very recently also with an N-acyl-mannosamine.^20^ The azide functional group has been most useful for these types of studies as it is not found in mammals and it undergoes a specific 1,3-dipolar cycloaddition with alkynes, either catalysed by copper or promoted by ring strain. The azide group has also been used for metabolic labelling of bacterial lipopolysaccharides.^23^ New bioorthogonal chemistries that are compatible with the metabolic glycan labelling strategy and are additionally orthogonal to the azide reporter group, are urgently required to further facilitate the study of cell-surface glycosylation.

The isonitrile (or isocyano) group is neutral overall and consists of just two atoms. Isonitriles are stable at biologically relevant pH values and display no appreciable toxicity in mammals.^24^–^26^ Like the widely utilised azide group, isonitriles are not found in humans, though they are found in some natural products from various marine and terrestrial sources.^27^, ^28^ Isonitriles undergo a [4+1] cycloaddition with tetrazines, which have already been used extensively as bioorthogonal reaction partners for *trans*-cyclooctenes.^29^, ^30^ With many primary and secondary isonitriles, a tautomerisation to an imine occurs, and in aqueous solution this imine hydrolyses rapidly. However, we have shown that adducts with tertiary isonitriles, which cannot tautomerise, are remarkably stable and that, with a primary 3-isocyanopropionyl ester, the imine tautomerises further to a conjugated enamine, which is slow to hydrolyse.^31^ The rates of reaction of a dipyridyltetrazine probe with primary (3-isocyanopropionate) and tertiary isonitriles were 0.12 and 0.57 m^−1^ s^−1^, and the adducts have half-lives of 16 and 63 h, respectively, in aqueous solution.^31^ Thus, in terms of reactivity and stability of the conjugate, the tertiary isonitrile would be a more attractive choice for a bioorthogonal ligation, but its greater size might prevent its metabolic incorporation into glycans by the host cell's enzymes.

We hypothesised that, owing to its small size, stability and reactivity with tetrazine, the isonitrile group would be an ideal candidate for a chemical reporter suitable for glycan imaging. In this paper, we present a proof-of-principle study in which isonitrile-bearing sugars are incorporated into cell surfaces and labelled by a bioorthogonal isonitrile–tetrazine click reaction.

First we checked that both primary and tertiary isonitriles are bioorthogonal by incubating them with 10 mm glutathione for 24 h at pH 7.4 and 37 °C. No reaction was detected by ^1^H NMR spectroscopy (see the Supporting Information).

To begin the study itself, primary and tertiary isonitrile derivatives of glucosamine, galactosamine and mannosamine were synthesised (Scheme 1). Ac_4_GlcN-*n*-Iso and Ac_4_GalN-*n*-Iso were synthesised by 1-ethyl-3-(3-(dimethylamino)propyl)carbodiimide hydrochloride (EDC) coupling of the corresponding O-peracetylated, free-amine sugars with 3-formamidopropanoic acid followed by dehydration with phosphorus oxychloride. Ac_4_ManN-*n*-Iso was produced by a similar coupling reaction between mannosamine and 3-(formamido)propanoic acid, followed by peracetylation with acetic anhydride and pyridine, and then dehydration of the formamide. The tertiary isonitrile sugars Ac_4_GlcN-*t*-Iso, Ac_4_GalN-*t*-Iso and Ac_4_ManN-*t*-Iso were formed in an analogous fashion by coupling 2-isocyano-2-methylpropanoic acid with the same amino sugar derivatives (see the Supporting Information for details).

**Scheme 1:**
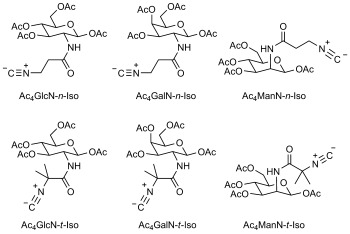
Structures of the six isonitrile sugars used in this study.

A two-step labelling procedure is normally used to detect incorporation of chemical reporter groups into cell-surface glycans; the cell surface is initially biotinylated by treatment with the chemical reporter. The biotinylated surface can then be visualised by using fluorescently labelled neutravidin followed by confocal microscopy or flow cytometry. This approach improves the signal-to-background ratio (SBR), as it allows one to compensate for the slow rate of the first bioorthogonal reaction by delivering a high concentration of non-fluorogenic probe. The fluorescent component can then be delivered at a much lower concentration, due to the rapid kinetics of biotin–neutravidin binding, thus minimising the background signal that arises from nonspecific binding. To apply this approach, a tetrazine-biotin conjugate (Tz–biotin, Scheme 2 A) was synthesised (see the Supporting Information).

**Scheme 2:**
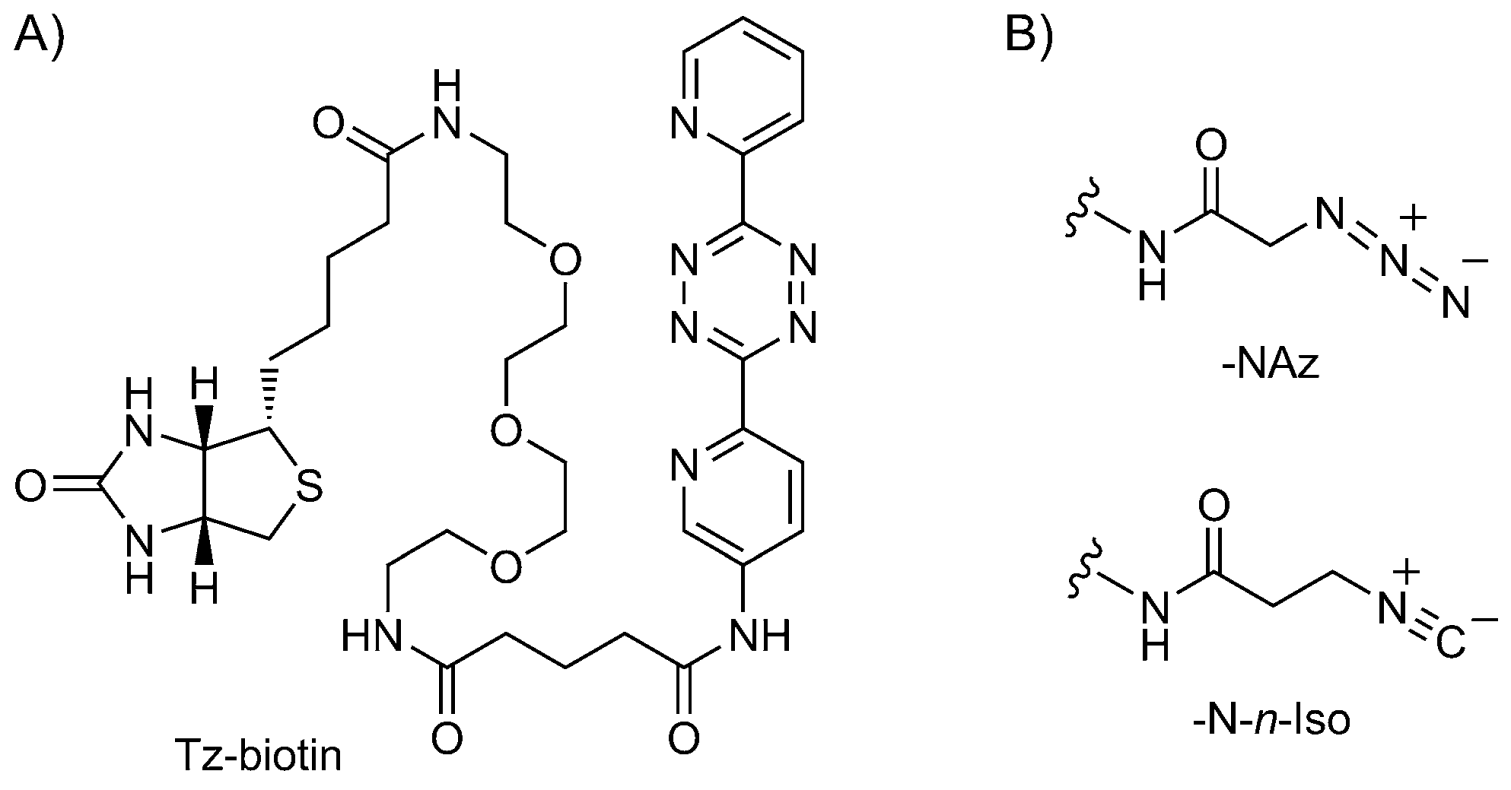
A) Structure of the tetrazine-biotin conjugate. B) Comparison of the shapes of the -NAz and N-*n*-Iso groups

Lewis lung carcinoma (LL2) cells were seeded at a density of 2×10^4^ cells cm^−2^ and incubated for 24 h. The cells were then exposed to either the vehicle (DMSO, <0.25% *v*/*v*) or isonitrile sugar at 200 μm and incubated for a further 24 h. The cells were harvested and treated first with 100 μm Tz–biotin for 30 min then, after a series of washes with ice-cold buffer, with neutravidin-DyLight680 for 15 min. After a final set of washes, the cells were analysed by flow cytometry (Figure 1).

**Figure 1:**
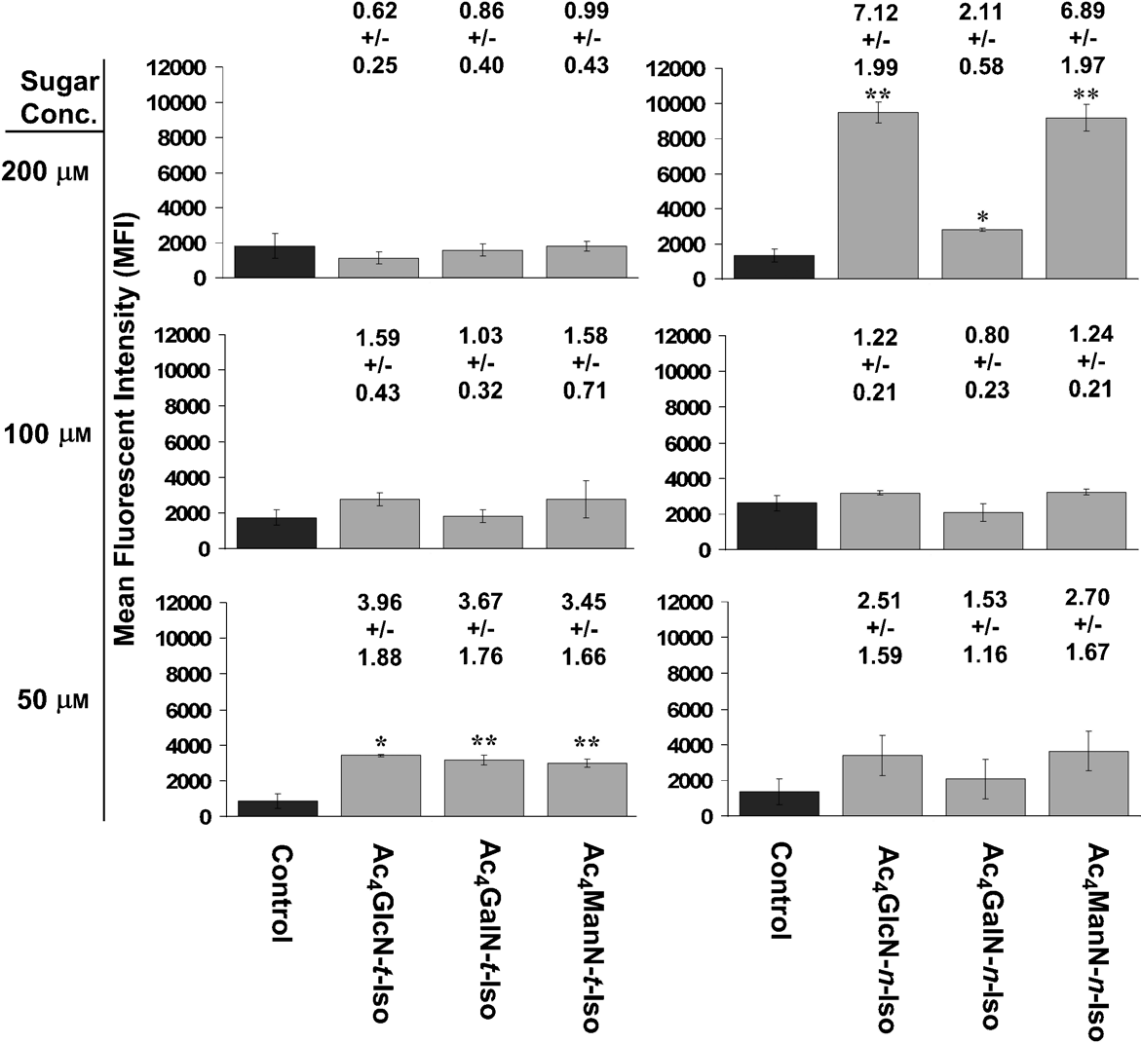
Flow cytometry results from the two-step labelling procedure. LL2 cells were exposed to either vehicle (DMSO, <0.25% *v*/*v*, control) or one of the isonitrile sugars. The mean fluorescence intensity (MFI) of the cells after the labelling procedure is shown. Error bars represent standard deviation of three repeats, 10 000 events per repeat (see Figures S1 and S2 for details). Numbers above bars are the SBRs (sugar MFI/control MFI) and the associated error. *P<0.05, **P<0.005, differences between control and sugar MFI were significant, two-tailed *t*-test.

There was no significant difference in the mean fluorescence intensity (MFI) of the tertiary isonitrile sugar pulsed cells and the control cells. As it has previously been shown that the click reaction between a similar tetrazine probe and a tertiary isonitrile is bioorthogonal, the sugars had either not been delivered to the cell surface as glycans or the isonitrile group had been degraded in the process. However, with the primary isonitrile sugars, the pulsed cells showed significant SBRs of 7.1(±2.0) for Ac_4_GlcN-*n*-Iso, 6.9(±2.0) for Ac_4_ManN-*n*-Iso and 2.1(±0.6) for Ac_4_GalN-*n*-Iso. Thus it appears that not only are the 3-isocyanopropionyl (*n*-Iso)-substituted sugars accepted as substrates for the glycan biosynthetic enzymes but also the isonitrile group is sufficiently metabolically stable during the metabolic processing to be expressed intact on the cell surface.

A very interesting aspect of these results is the relatively high incorporation of the glucosamine derivative and low incorporation of the galactosamine derivative. Bertozzi and co-workers have previously shown that it is the other way round with azido sugars, and *N*-azidoacetyl-glucosamine (GlcNAz) is poorly incorporated into the cell-surface glycans of human cell lines,^32^ whereas GalNAz is well incorporated, and we have had the same experience. It has been shown that the action of UDP-GlcNAc-pyrophosphorylase on GlcNAz 1-phosphate is less efficient than on GlcNAc 1-phosphate, the native substrate.^33^, ^34^ It has been speculated, however, that UDP-GlcNAz should still accumulate due to the lack of feedback inhibition in the hexosamine salvage pathway, and so the poor incorporation of GlcNAz into cell-surface proteins might also be due to other factors such as inefficient transport, diversion into other metabolic pathways or low tolerance of GlcNAc transferases for UDP-GlcNAz.^33^

Next, the flow cytometry experiments were repeated with 100 and 50 μm of the isonitrile sugars (Figure 1). Whereas the incorporation levels of tertiary isonitrile sugars only increased slightly at a concentration of 100 μm, the levels significantly increased at a concentration of 50 μm, with SBRs of 4.0(±1.9) for Ac_4_GlcN-*t*-Iso, 3.7(±1.8) for Ac_4_GalN-*t*-Iso and 3.5(±1.7) for Ac_4_ManN-*t*-Iso. This increase is probably due to the decreased toxicity of the tertiary isonitrile sugars at lower concentrations (see below). Interestingly, the incorporation of primary isonitrile sugars at concentrations of 100 or 50 μm was considerably lower than at 200 μm, with SBRs of 2.5(±1.6), 1.5(±1.2) and 2.7(±1.7) for Ac_4_GlcN-*n*-Iso, Ac_4_GalN-*n*-Iso and Ac_4_ManN-*n*-Iso, respectively, at 50 μm.

The structural differences between GlcN-*n*-Iso and GlcNAz are subtle, as both side chains are of similar length, they are both neutral overall and they have the same geometry with a linearly arranged set of three atoms at the terminus (Scheme 2 B). The first nitrogen atom of the azide group is replaced by a methylene group and consequently the isonitrile is slightly more bulky, but it would be surprising if this small difference had a major effect. A more significant difference could be the ability of the first nitrogen atom of the azide group to participate in hydrogen bonding, which is not possible for the methylene group of the primary isonitrile. Nevertheless, the results show that bioorthogonal glycan labelling by isonitrile–tetrazine click chemistry works well with the primary sugars at high concentrations (200 μm) and, to a lesser extent, with the tertiary sugars at low concentrations (50 μm). This new bioorthogonal ligation could facilitate important advances in the understanding of protein glycosylation.

To confirm our findings, quantitative epifluorescence microscopy images of labelled cells were obtained. As little labelling was observed with the tertiary isonitrile sugars, only the primary isonitrile sugars were used (Figure 2). As expected, the fluorescent labelling was restricted to the cell surfaces. The increases in fluorescence of the pulsed over the unpulsed cells (rows A and C) were 2.7-, 5.6- and 5.8-fold for Ac_4_GalN-*n*-Iso, Ac_4_GlcN-*n*-Iso and Ac_4_ManN-*n*-Iso, respectively; this closely correlates with the values obtained by flow cytometry.

**Figure 2:**
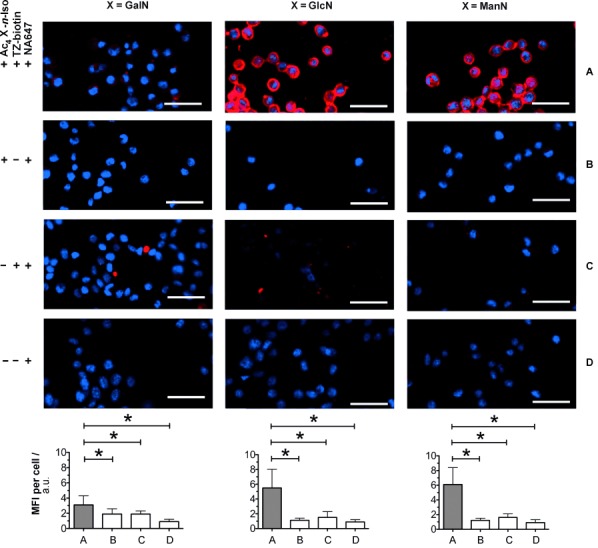
Fluorescence imaging and cytometry of LL2 cells metabolically labelled with isonitrile sugars. Cells were incubated with 200 μm of each of the isonitrile sugars (Ac_4_Gal-*n*-Iso, Ac_4_Glc-*n*-Iso or Ac_4_Man-*n*-Iso) or with vehicle for 24 h at 37 °C, then with Tz–biotin (300 μm) or vehicle for 30 min at 37 °C and then with NA647 (50 μg mL^−l^) and DAPI nuclear stain for 15 min at RT. Red: NA647, Blue: DAPI. Scale bars: 100 μm. Images were analysed by quantitative imaging cytometry, and the MFI (bottom) was calculated for each experimental group (A to D) corresponding to the different combinations of the three reagents used. Values plotted in the charts are mean±SD (3500–5000 events counted per cell culture well). *P<0.0005, differences between groups were significant, two-tailed *t*-test.

In order to understand why higher concentrations of the tertiary isonitrile (*t*-Iso) sugars give lower labelling levels, their effect on cell growth was examined. LL2 cells were grown in a 96-well microplate, and the level of confluence was measured at three hour intervals over the course of one week (Figure S3). The sugars were administered at 200 μm after 24 h and dramatically retarded cell growth and caused a clear change in morphology (Figure S4). Cells shrank and became more circular after 24 h, thus accounting for the slight reduction in confluence. During the flow cytometry experiment (Figure 1), viable cells were selected on the basis of high NADH autofluorescence and low fluorescence from the cell-impermeable nucleic acid stain Sytox Green. With the *t*-Iso sugars, cell viability (64–82 %, Figure S5) was similar to that of the control (86 %), thus suggesting that these sugars are cytostatic rather than cytotoxic. This cytostatic effect of the tertiary isonitrile sugars might be the reason for the lack of sugar incorporation observed in Figure 1.

A smaller effect was observed for cells treated with the primary isonitrile sugars. The cells did grow for about 24 h after sugar-treatment but more slowly than the control cells, and growth ceased after longer periods. The percentage of viable cells was the same for Ac_4_GlcN-*n*-Iso- and Ac_4_ManN-*n*-Iso-treated cells as for control cells (70–77 % vs. 84 %, Figures S1 and S5). Cells exposed to Ac_4_GalN-*n*-Iso were less viable (59 %) than the control cells. This modest toxicity of Ac_4_GalN-*n*-Iso could to some extent explain why incorporation levels were lower than for the corresponding Glc and Man derivatives. Additionally, the toxicity experiments were repeated with sugar concentrations of 100 and 50 μm (Figure S3). The cytostatic effect of the tertiary isonitrile sugars was much reduced at lower concentrations, particularly in the case of Ac_4_ManN-*t*-Iso at 50 μm, which, after 24 h, only reduced cells to ≍70 % of the confluence of the control cells. Large increases in growth were observed at the lower concentrations of Ac_4_ManN-*n*-Iso and especially Ac_4_GalN-*n*-Iso, but growth was only modestly improved at the lower concentrations of Ac_4_GlcN-*n*-Iso.

To test the orthogonality of isonitrile–tetrazine click chemistry with azide–alkyne click chemistry, a solution of 1-pentyl isocyanide and tetramethoxydibenzocyclooctyne^35^ (TMDIBO; each 20 mm) in CD_3_CN/D_2_O (1:1) was incubated at 37 °C for 24 hours. NMR spectroscopy then showed that no reaction had occurred. The same was found for mixtures of *tert*-butyl isonitrile and TMDIBO and of benzyl azide with both isonitriles (see the Supporting Information). As it is known that dibenzocyclooctynes do not react with tetrazines,^36^, ^37^ we can say that isonitrile–tetrazine click chemistry is orthogonal to azide–alkyne chemistry, provided dibenzocyclooctynes are used.^38^

In conclusion, we have shown that isonitrile–tetrazine click chemistry can be used to metabolically label cell-surface glycans. Ac_4_GlcN-*n*-Iso and Ac_4_ManN-*n*-Iso show good incorporation whereas Ac_4_GalN-*n*-Iso only gives a modest twofold increase in fluorescence over untreated cells. We have shown that the primary isonitrile sugars Ac_4_GlcN-*n*-Iso and Ac_4_ManN-*n*-Iso are not toxic to cells, even at high concentrations, and that cells continue to grow for the first 24 hours after treatment (the length of the incubations used in the labelling experiments). We have also shown that Ac_4_GlcN-*t*-Iso, Ac_4_GalN-*t*-Iso and Ac_4_ManN-*t*-Iso are also incorporated, although only at lower concentrations of sugar, giving SBR's of around threefold. This study expands the chemical toolbox for metabolic glycan labelling, which until now has been heavily reliant on azide–alkyne chemistry. Finally, as isonitrile–tetrazine chemistry is orthogonal to the popular azide–alkyne chemistry, simultaneous differential multi-sugar imaging is a real possibility with this chemistry.

While this manuscript was in preparation, two-sugar glycan imaging by using a cyclopropene/tetrazine cycloaddition^20^ and (even more recently) a linear alkene/tetrazine cycloaddition,^18^ both combined with azide–alkyne cycloaddition for the second sugar, have been reported.

## Supplementary material

As a service to our authors and readers, this journal provides supporting information supplied by the authors. Such materials are peer reviewed and may be re-organized for online delivery, but are not copy-edited or typeset. Technical support issues arising from supporting information (other than missing files) should be addressed to the authors.
