# Supplementary material for: Metabolic Glycan Imaging by Isonitrile–Tetrazine Click Chemistry
Source: Chembiochem. 2013 May 13;14(9):1063–7. doi: 10.1002/cbic.201300130 (PMC3743162; doi:10.1002/cbic.201300130)
Supplement: Supplementary file 1 [file cbic0014-1063-SD1.pdf]

CHEMBIOCHEM

Supporting Information

© Copyright Wiley-VCH Verlag GmbH & Co. KGaA, 69451 Weinheim, 2013

Metabolic Glycan Imaging by Isonitrile–Tetrazine Click Chemistry

Shaun Stairs,[a] André A. Neves,[b] Henning Stöckmann,[a] Yelena A. Wainman,[a] Heather Ireland-Zecchini,[b] Kevin M. Brindle,[b] and Finian J. Leeper*[a]

cbic_201300130_sm_miscellaneous_information.pdf

Supplementary Information For 'Metabolic Glycan Imaging By Isonitrile-Tetrazine Click Chemistry'

Shaun Stairs, Andre A. Neves, Henning Stöckmann, Yelena A. Wainman, Heather Ireland-Zecchini, Kevin M. Brindle and Finian J. Leeper

S1 – General Information
S2 – Synthesis of Reagents
S3 – Biological Procedures
S4 – Further Data
S5 – References
S6 – NMR Spectra

S1

General Information

^{1}H-NMR spectra were recorded with an internal deuterium lock for the residual protons in $CDCl_3$ (δ 7.26) at ambient probe temperature on a Bruker DRX400 instrument. Data are presented as follows: chemical shift (in ppm), integration, multiplicity (s=singlet, d=doublet, t=triplet, q=quartet, qu=quintet, m=multiplet, br=broad, app=apparent), coupling constants in Hz and interpretation. Coupling constants are reported as they appear in the spectra. Assignments were determined either on the basis of unambiguous chemical shift data or coupling patterns, COSY experiments or by analogy to fully interpreted spectra for related compounds. ^{13}C-NMR spectra were recorded with an internal deuterium lock for $CDCl_3$ (δ 77.0) at ambient probe temperature. Chemical shifts are given in ppm. Infrared spectra were recorded on a Perkin Elmer Spectrum One (FT-IR) spectrophotometer. Wavelengths of maximum absorbance (ν_{max}) are quoted in wavenumbers (cm^{-1}). High resolution mass spectra (HRMS) were recorded by the Chemical Laboratory Mass Spec Service and were recorded on a Waters LCT Premier TOF mass spectrometer with electrospray and modular Lockspray interface. The parent ion $[MH]^+$, $[M+NH_4]^+$ or $[M+Na]^+$ is quoted. All solvent mixtures are reported as % vol/vol unless otherwise stated. Analytical thin layer chromatography (TLC) was carried out on Merck Kieselgel 60 F_{254} plates with visualisation by ultra violet light (254 nm), potassium permanganate and/or PMA/$Ce(SO_4)_2$ dip. Flash column chromatography was carried out using Merck Kieselgel 60 (230-400 mesh) under a positive pressure using distilled solvents and the procedure includes the subsequent evaporation of solvents. All evaporation of solvents was performed under reduced pressure. Reagents and solvents were purified using standard means. Dichloromethane (DCM), acetonitrile (MeCN), methanol (MeOH), toluene (PhMe) and hexane were distilled from calcium hydride (CaH_2) under an argon atmosphere; tetrahydrofuran (THF) and diethyl ether (Et_2O) were distilled from potassium/benzophenone or sodium/benzophenone, respectively, and stored under an argon atmosphere. All other chemicals were used as received unless otherwise noted. All other extractive procedures were performed using distilled solvents and all aqueous solutions used were saturated. Unless otherwise stated, all non-aqueous reactions were carried out under an argon atmosphere using anhydrous conditions and oven-dried glassware. Standard techniques were employed for handling air-sensitive materials.

Stability of isonitriles in the presence of biologically relevant functional groups
Stock solutions of glutathione, n-pentyl isocyanide and t-butyl isocyanide were prepared in CD_3CN/D_2O (1:1) at 20 mM. The glutathione solution was basified to pH 7.4. The following mixtures were prepared:
(a) 1:1 molar equivalent of glutathione/pentyl isocyanide at 10 mM (700 μL total)
(b) 1:1 molar equivalent of glutathione/t-butyl isocyanide at 10 mM (700 μL total)
(c) Individual controls (glutathione and isonitriles) diluted to 10 mM.
The NMR tubes (a) and (b) were incubated at 37 °C for 24 h. ^{1}H NMR spectra were run at 2 and 24 h.

S2

Synthesis of Reagents

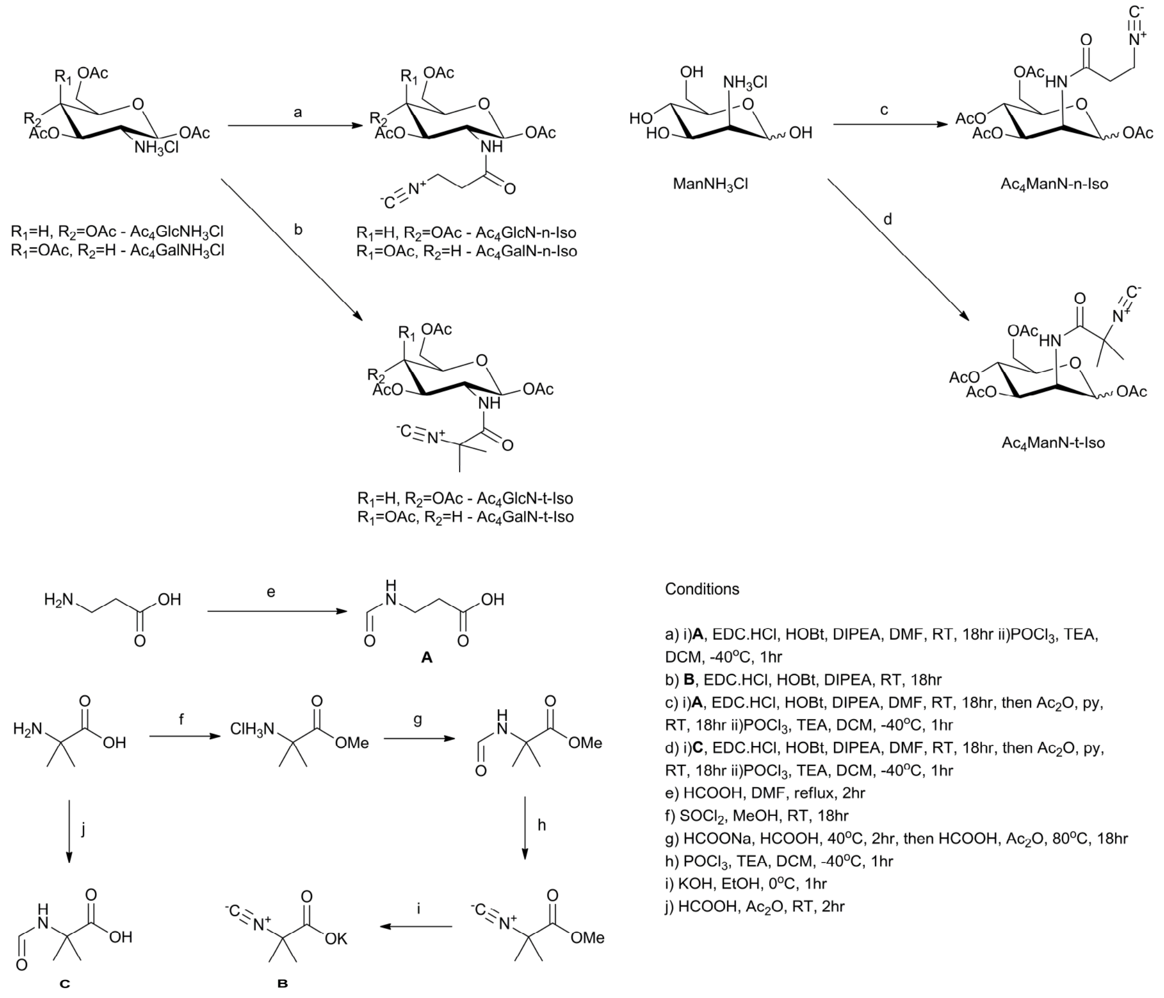

Ac_4GlcNH_3Cl and Ac_4GalNH_3Cl were synthesised according to the procedure used by Faroux-Corlay[1] and co-workers. **C** (2-formamido-2-methylpropanoic acid) was synthesised by a previously reported method[6]

3-Formamidopropanoic acid (**A**)
3-Aminopropionic acid (10 g, 112 mmol) and formic acid (8.78 g, 191 mmol) were heated at reflux in DMF (51 ml) for 2 hr. The solvent was evaporated to give fine white crystals which were suspended in ether, filtered, washed and dried to give the title compound (13.14 g, 112 mmol, 100%).
M.p. 72-74 °C lit, 74-75 °C[7]
^{1}H-NMR (400 MHz, DMSO) δ 8.04 (1H, br s, NH, 7.95 (1H, br s, NCHO), 3.26 (2H, app q, J = 6.7 Hz, $NHC\underline{H}_2CH_2$), 2.37 (2H, t, J = 6.7 Hz, $CH_2C\underline{H}_2COOH$); values agreed with literature[6]

Potassium 2-isocyano-2-methylpropanoate (**B**)

Methyl 2-isocyano-2-methylpropanoate (1 g, 7.94 mmol), synthesised according to procedures of Pirali et al.,[2] was dissolved in ethanol (15.9 ml) and cooled to 0 °C. Potassium hydroxide (445 mg, 7.94 mmol)

was added and the solution was stirred at 0 ^oC for 1 hr and then evaporated. The product was precipitated with ether, filtered, washed and dried to give the title compound as white crystals (563 mg, 3.75 mmol, 47%).

M.p. 169-172 ^oC

IR (cm^{-1}) ν_{max}: 2983 (CH), 2143 (NC), 1610 (COOK)

^{1}H-NMR (400 MHz, D_2O) δ 1.47 (6H, s).

^{13}C-NMR (125 MHz, D_2O) δ 174.7, 161.4 (t, 1:1:1), 55.1, 23.9.

HRMS *m/z*: $[M]^-$ calcd for $C_5H_6NO_2$, 130.0510; found, 130.0531

Tetra-O-acetyl-N-(3-isocyanopropanoyl)-D-glucosamine (Ac_4GlcN-n-Iso)

Ac_4GlcNH_3Cl (50 mg, 0.130 mmol), **A** (46 mg, 0.391 mmol), EDC.HCl (125 mg, 0.651 mmol), HOBt (100 mg, 0.651 mmol) and Et_3N (54 μl, 0.391 mmol) were stirred in DMF (1 ml) overnight. The solvent was evaporated under reduced pressure and the residue was rapidly eluted through a column of silica gel (approx. 15 ml) with 2% MeOH/DCM to remove EDC impurities. Fractions were concentrated, dissolved in DCM (1 ml), cooled to -40 ^oC and then treated successively with Et_3N (27.3 μl, 0.195 mmol) and phosphorus oxychloride (9.3 μl, 0.059 mmol). After 1 hr the reaction was quenched with saturated aq. $NaHCO_3$ and extracted with EtOAc. The combined extracts were washed with water, saturated aq. $NaHCO_3$ and brine, dried over $MgSO_4$ and evaporated. The product was purified by silica column chromatography, eluting with 2% MeOH/DCM, to yield the title compound as a colourless oil (20.1 mg, 0.047 mmol, 36%).

IR (cm^{-1}) ν_{max}: 3304 (NH), 2151 (NC), 1745 (OAc), 1669 (NHCO)

^{1}H-NMR (400 MHz, $CDCl_3$) δ 6.07 (1H, d, *J* 9.6, NH), 5.76 (1H, d, *J* 8.8, C1), 5.25 (1H, t, *J* 10, C3), 5.16 (1H, t, *J* 10, C4), 4.37 (1H, app q, *J* 9.6, C2), 4.31 (1H, dd, *J* 12.5, 4.7, C6a), 4.16 (1H, dd, *J* 12.5, 2.1, C6b), 3.89 (1H, m, C5), 3.73 (2H, m, CH_2), 2.50 (2H, t, *J* 6.2, CH_2), 2.16, 2.12, 2.10 and 2.08 (4 x 3H, s, Ac).

^{13}C-NMR (125 MHz, $CDCl_3$) δ 172.0, 171.1, 170.0, 169.7, 169.0, 157.8, 92.8, 73.2, 72.9, 68.3, 62.1, 53.5, 38.1, 36.5, 21.4, 21.3, 21.2, 21.0.

HRMS *m/z*: $[M+H]^+$ calcd for $C_{18}H_{25}N_2O_{10}$, 429.1509; found, 429.1521.

Tetra-O-acetyl-N-(3-isocyanopropanoyl)-D-galactosamine (Ac_4GalN-n-Iso).

This was synthesised from Ac_4GalNH_3Cl by the above method to give the title compound as a colourless oil (31.5 mg, 0.074 mmol, 57%).

IR (cm^{-1}) ν_{max}: 3336 (NH), 2152 (NC), 1745 (OAc), 1688 (NHCO)

^{1}H-NMR (400 MHz, $CDCl_3$) δ 6.15 (1H, d, *J* 9.3, NH), 5.79 (1H, d, *J* 8.8, C1), 5.41 (1H, d, *J* 3.4, C4), 5.18 (1H, dd, *J* 11.3, 3.4, C3), 4.52 (1H, app dt, *J* 11.3, 9.3, C2), 4.15 (3H, m, C5, C6), 3.73 (2H, t, *J* 6.0, CH_2), 2.51 (2H, t, *J* 6.0, CH_2), 2.21, 2.17, 2.08 and 2.06 (4 x 3H, s, OAc).

^{13}C-NMR (125 MHz, $CDCl_3$) δ 171.3, 170.9, 170.6, 170.2, 169.3, 157.7, 93.1, 72.1, 70.6, 66.7, 61.8, 50.2, 36.7, 33.6, 21.5, 21.3, 21.1, 21.1.

HRMS *m/z*: $[M+H]^+$ calcd for $C_{18}H_{25}N_2O_{10}$, 429.1509; found, 429.1518.

Tetra-O-acetyl-N-(3-isocyanopropanoyl)-D-mannosamine (Ac_4ManN-n-Iso).

Mannosamine.HCl (100 mg, 0.463 mmol), **A** (57.2 mg, 0.489 mmol), EDC.HCl (123 mg, 0.642 mmol), HOBt (98 mg, 0.725 mg) and Et_3N (64 μl, 0.459 mmol) were stirred in DMF (2.14 ml) overnight. The solvent was evaporated under reduced pressure and the residue was dissolved in pyridine (3 ml), cooled to 0 ^oC and acetic anhydride (1.7 ml) was added. The mixture was stirred for 1 hr then warmed to RT and stirred overnight. It was evaporated and then co-evaporated with water (3x) and toluene (3x) then rapidly eluted through a column of silica gel (approx 30 ml) with 2% MeOH/DCM to remove EDC impurities. Fractions were concentrated, dissolved in DCM (1 ml), cooled to -40 ^oC and then treated successively

with Et_3N (112 µl, 0.780 mmol) and phosphorus oxychloride (39 µl, 0.404 mmol). After 1 hr the reaction was quenched with saturated aq. $NaHCO_3$ and extracted with EtOAc. The combined extracts were washed with water, saturated aq. $NaHCO_3$, and brine, dried over $MgSO_4$ and evaporated. The product was purified by silica column chromatography, eluting with 2% MeOH/DCM, to yield the title compound as a colourless oil (52 mg, 0.121 mmol, 26%).
IR (cm^{-1}) ν_{max}: 3263 (NH), 2152 (NC), 1743 (OAc), 1675 (NHCO)
^{1}H-NMR (400 MHz, $CDCl_3$) δ 6.08 (1H, d, *J* 8.9, NH), 6.08 (1H, d, *J* 1.8, C1), 5.38 (1H, dd, *J* 10.1, 4.4, C3), 5.21 (1H, t, *J* 10.1, C4), 4.70 (1H, ddd, *J* 8.9, 4.4, 1.8, C2), 4.33 (1H, dd, *J* 12.2, 4.9, C6a), 4.10 (2H, m, C5, C6b), 3.77 (2H, m, CH_2), 2.71 (2H, m, CH_2), 2.22, 2.14, 2.10 and 2.05 (4 x 3H, s, OAc).
^{13}C-NMR (125 MHz, $CDCl_3$) δ 171.1, 170.5, 170.1, 169.0, 168.5, 157.9, 91.9, 70.6, 69.0, 65.9, 62.5, 50.0, 37.8, 36.1, 21.3, 21.2, 21.2, 21.1
HRMS *m/z*: $[M+H]^+$ calcd for $C_{18}H_{25}N_2O_{10}$, 429.1509; found, 429.1527.

Tetra-O-acetyl-N-(2-isocyano2-methylpropanoyl)-D-glucosamine (Ac_4GlcN-t-Iso).
Ac_4GlcNH_3Cl (50 mg, 0.130mmol), **B** (59 mg, 0.391 mmol), EDC.HCl (125 mg, 0.651 mmol), HOBt (100 mg, 0.651 mmol) and Et_3N (18 µl, 0.13 mmol) were stirred in DMF (1 ml) overnight. The solvent was evaporated under reduced pressure and the residue was purified by silica column chromatography, eluting with 2% MeOH/DCM, to yield the title compound as a colourless oil (10.2 mg, 0.023 mmol, 18%).
IR (cm^{-1}) ν_{max}: 3359 (NH), 2132 (NC), 1749 (OAc), 1694 (NHCO)
^{1}H-NMR (400 MHz, $CDCl_3$) δ 6.83 (1H, d, *J* 9.1, NH), 5.92 (1H, d, *J* 8.6, C1), 5.42 (1H, app t, *J* 9.3, C3), 5.18 (1H, t, *J* 9.6, C4), 4.32 (1H, dd, *J* 12.5, 4.9, C6a), 4.17 (2H, m, C6b, C2), 3.91 (1H, m, C5), 2.14, 2.12, 2.09 and 2.08 (4 x 3H, s, Ac), 1.62 and 1.61 (2 x 3H, s, CH_3).
^{13}C-NMR (125 MHz, $CDCl_3$) δ 171.3, 171.1, 169.9, 169.7, 169.5, 161.2, 92.4, 73.4, 72.2, 68.2, 62.1, 61.5, 54.2, 28.0 (2xMe), 21.1, 21.1, 20.9, 20.9
HRMS *m/z*: $[M+H]^+$ calcd for $C_{19}H_{27}N_2O_{10}$, 443.1660; found, 443.1674

Tetra-O-acetyl-N-(2-isocyano2-methylpropanoyl)-D-galactosamine (Ac_4GalN-t-Iso).
This was synthesised from Ac_4GalNH_3Cl by the above method to give the title compound as a colourless oil (15 mg, 0.034 mmol, 26%).
IR (cm^{-1}) ν_{max}: 3360 (NH), 2134 (NC), 1748 (OAc), 1695 (NHCO)
^{1}H-NMR (400 MHz, $CDCl_3$) δ 6.72 (1H, d, *J* 9.3, NH), 5.93 (1H, d, *J* 8.6, C1), 5.44 (1H, d, *J* 3.4, C4), 5.35 (1H, dd, *J* 11.2, 3.4, C3), 4.39 (1H, app dt, *J* 11.2, 9.1, C2), 4.15 (3H, m, C5, C6), 2.20, 2.15, 2.08 and 2.05 (3H, s, Ac), 1.62 (6H, s, CH_3).
^{13}C-NMR (125 MHz, $CDCl_3$) δ 170.8, 170.8, 170.5, 170.1, 169.6, 160.8, 92.8, 72.3, 70.2, 66.8, 61.7, 61.5, 51.0, 28.1, 28.0, 21.2, 21.1, 21.1, 20.9
HRMS *m/z*: $[M+H]^+$ calcd for $C_{19}H_{27}N_2O_{10}$, 443.1660; found, 443.1649

Tetra-O-acetyl-N-(2-isocyano2-methylpropanoyl)-D-mannosamine (Ac_4ManN-t-Iso)
Mannosamine.HCl (70 mg, 0.322 mmol), **C** (48.3 mg, 0.343 mmol), EDC.HCl (86.1 mg, 0.448 mmol), HOBt (68.6 mg, 0.448 mmol) and Et_3N (44.8 µl, 0.322 mmol) were stirred in DMF (1.5 ml) overnight. The solvent was evaporated under reduced pressure and the residue was dissolved in pyridine (1 ml), cooled to 0 °C and treated with acetic anhydride (1.12 ml). The mixture was stirred for 1 hr then warmed to RT and stirred overnight. The mixture was evaporated and then co-evaporated with water (3x) and toluene (3x) then rapidly eluted through a column of silica gel (approx 20 ml) with 2% MeOH/DCM to remove EDC impurities. Fractions were evaporated, dissolved in DCM (1 ml), cooled to -40 °C and then treated successively with Et_3N (63 µl, 0.45 mmol) and phosphorus oxychloride (13.4 µl, 0.14 mmol). After 1 hr the reaction was quenched with saturated aq. $NaHCO_3$ solution and extracted with EtOAc. The combined extracts were washed with water, saturated aq. $NaHCO_3$, and brine, dried over $MgSO_4$ and evaporated. The product was purified by silica column chromatography, eluting with 2% MeOH/DCM, to yield the title compound as a colourless oil (8.9 mg, 0.02 mmol, 6.3%).

IR (cm^{-1}) ν_{max}: 3247 (NH), 2130 (NC), 1743 (OAc), 1690 (NHCO)
^{1}H-NMR (400 MHz, $CDCl_3$) showed a 1:4 mixture of anomers: δ (major anomer) 6.85 (1H, d, *J* 9.3, NH), 6.13 (1H, d, *J* 1.9, C1), 5.37 (1H, dd, *J* 10.1, 3.9, C3), 5.34 (1H, t, *J* 10.1, C4), 4.64 (1H, ddd, *J* 9.3, 3.9, 1.9, C2), 4.26 (1H, dd, *J* 12.5, 3.1, C6a), 4.15 (1H, dd, *J* 12.5, 2.3, C6b), 4.09 (1H, br app d, *J* 10.1, C5), 2.23, 2.16, 2.09 and 2.00 (4 x 3H, s, OAc); (minor anomer) 6.96 (1H, d, *J* 8.8, NH), 5.95 (1H, d, *J* 1.6, C1), 5.27 (1H, t, *J* 9.5, C4), 5.11 (1H, dd, *J* 9.5, 3.9, C3), 7.73 (1H, ddd, *J* 8.8, 3.9, 1.6, C2), 4.27 (1H, dd, *J* 12.5, 5.2, C6a), 4.21 (1H, dd, *J* 12.5, 2.6, C6b), 3.87 (1H, br app dt, *J* 9.5, 2.9, C5), 2.16, 2.16, 2.09 and 2.03 (4 x 3H, s, OAc).
^{13}C-NMR (125 MHz, $CDCl_3$) δ (major anomer) 171.3, 170.4, 169.8, 169.7, 168.5, 162.0, 91.6, 70.6, 69.5, 64.9, 61.8, 61.6, 50.0, 28.5, 27.8, 21.3, 21.2, 21.0, 21.0; (minor anomer) 171.2, 170.3, 169.8, 169.7, 168.7, 162.0, 90.5, 73.6, 71.7, 65.0, 61.9, 61.7, 50.4, 28.3, 28.0, 21.2, 21.1, 21.1, 21.0.
HRMS *m/z*: $[M+H]^+$ calcd for $C_{19}H_{27}N_2O_{10}$, 443.1666; found, 443.1677.

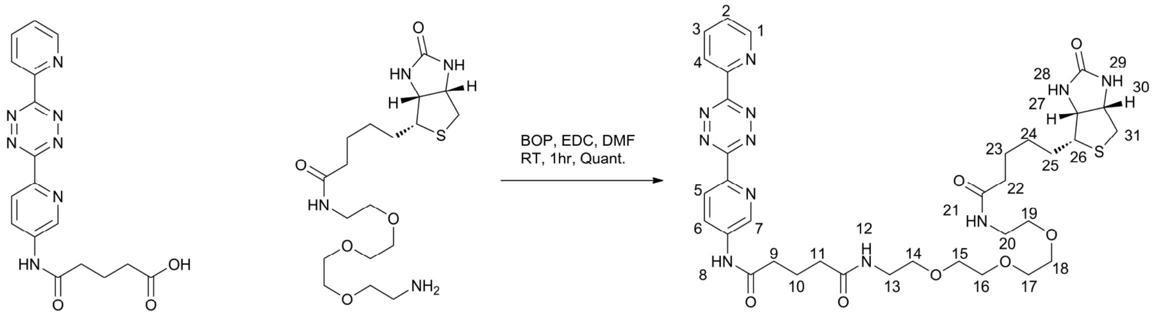

D **EZ-Link Amine-PEG3-Biotin** **TZ-Biotin**

5-Oxo-5-((6-(6-(pyridin-2-yl)-1,2,4,5-tetrazin-3-yl)pyridin-3-yl)amino)pentanoic acid (**D**) was synthesised according to procedures by Hansell *et al.*[3] EZ-Link Amine-PEG3-Biotin was purchased from Thermo Scientific.

Tz-Biotin
EZ-Link Amine-PEG3-Biotin (12.7 mg, 0.03 mmol) and **D** (11.1 mg, 0.03 mmol) were dissolved in dry DMF (500 µl). BOP (20.2 mg, 0.046 mmol) and Hünig's base (53.1 µl, 0.304 mmol) were added and the mixture was stirred at RT for 1 hr. The solvent was evaporated under reduced pressure and the residue was purified by HPLC (C18 column, MeCN/H_2O) to give the title compound (22.9 mg, 0.03 mmol, quant.)
IR (cm^{-1}) ν_{max}: 3284.6 (NH), 1691.8 (NHCO), 1647.4 (NHCONH), 1582.7 (Ar), 1541.6 (Ar)
^{1}H-NMR (400 MHz, DMSO) δ 10.57 (1H, br s, 8), 9.05 (1H, d, *J* 2.6, 7), 8.94 (1H, app br d, *J* 4.7, 1), 8.62 (1H, d, *J* 8.7, 5), 8.59 (1H, d, *J* 8.1, 4), 8.43 (1H, dd, *J* 8.7, 2.6, 6), 8.15 (1H, td, *J* 8.1, 1.8, 3), 7.91 (1H, t, *J* 5.5, 12/21), 7.82 (1H, t, *J* 5.5, 12/21), 7.73 (1H, ddd, *J* 8.1, 4.7, 1.1, 2), 6.41 (1H, br s, 28), 6.35 (1H, br s, 29), 4.30 (1H, app br t, *J* 7.0, 30), 4.12 (1H, m, 27), 3.50 (8H, m, 15,16,17,18), 3.41 (2H, t, *J* 6.2, 19/14), 3.38 (2H, t, *J* 6.0, 19/14), 3.19 (4H, m, 13, 20), 3.08 (1H, m, 26), 2.81 (1H, dd, *J* 12.5, 5.2, 31a), 2.57 (1H, d, *J* 12.5, 31b), 2.44 (2H, t, *J* 7.5, 9/11), 2.18 (2H, t, *J* 7.5, 9/11), 2.05 (2H, t, *J* 7.5, 22), 1.85 (2H, app qn, *J* 7.5, 10), 1.60 (1H, m, 25a), 1.48 (3H, m, 23, 25b), 1.28 (2H, m, 24).
^{13}C-NMR (125 MHz, DMSO) δ 173.0, 172.6, 163.9, 163.6, 163.6, 151.5, 151.5, 151.1, 144.7, 142.1, 139.4, 138.7, 127.5, 127.0, 125.8, 125.1, 70.6, 70.5, 70.4, 70.0, 61.9, 60.1, 56.3, 41.3, 39.4, 39.3, 36.6, 36.0, 35.5, 31.8, 29.1, 28.9, 26.1, 23.0, 21.8, 14.7,
HRMS *m/z*: $[M+H]^+$ calcd for $C_{35}H_{48}N_{11}O_7S$, 766.3453; found, 766.3506.

S3

Biological Procedures

Cell Culture

Lewis Lung Carcinoma (LL2, from ATCC, Teddington, UK) cells were grown in a mixture of 90% DMEM (Dulbecco's Modified Eagle's Medium, Invitrogen, Paisley, UK) supplemented with 4.5 g L^{-1} glucose, 4 mM L-glutamine, 1 mM pyruvate and 10% FBS (fetal bovine serum, PAA laboratories, Yeovil, UK) and maintained in a 5% CO_2, water-saturated atmosphere at 37 ^{o}C.

Cell surface isocyanoglycan labeling and detection by flow cytometry

LL2 cells were incubated for 24 hr in medium containing 200, 100 or 50 µM of the desired isonitrile sugar. Control cells were grown in the presence of the vehicle (DMSO) but absence of isonitrile sugar. Additionally a second set of control cells were grown without isonitrile sugar or vehicle. The following procedure was followed for both sugar-pulsed and non-pulsed sets of cells. The medium was removed from the flasks and cells were washed with warm PBS (phosphate buffered saline; water, NaCl, KCl, Na_2HPO_4, KH_2PO_4; Fisher Scientific, Loughborough, UK). Trypsin-EDTA (0.25% trypsin and 1 mM EDTA in Hanks' Balanced Salt Solution without $CaCl_2$, $MgCl_2$ or $MgSO_4$, Invitrogen) was added to the flask (7% of the original volume) and the cells incubated at 37 ^{o}C for 4.5 min before warm complete DMEM was added. The contents of each flask were transferred to a centrifuge tube, centrifuged (700 g, 4 ^{o}C, 4 min), resuspended in cold FACS buffer (1% FBS in PBS) and transferred to 1.5 mL Eppendorf tubes. Cells were centrifuged and resuspended in 100 µL labeling buffer A (100 µM Tz-Biotin in FACS buffer containing 50 nM SYTOX Green, Invitrogen) or as a control 100 µL 50 nM SYTOX Green in FACS buffer. The Eppendorf tubes were incubated in a hot block with orbital shaking (450 rpm, 37 ^{o}C, 30 min). After 30 min the cells were washed three times with 700 µL ice cold FACS buffer. Cells were then suspended in 100 µl labeling buffer B (50 µg/ml NeutrAvidin-Dylight680, Invitrogen in FACS buffer) or 100 µl FACS buffer as a control. The Eppendorf tubes were incubated in a hot block with orbital shaking (450 rpm, 37 ^{o}C, 15 min) and then the cells were washed two times with 700 µL ice cold FACS buffer, filtered through a 50 µm cut-off membrane into flow cytometry tubes and kept on ice. Each sample was analysed by a flow cytometer (model LSRII, BD Oxford, UK) using 10,000 events. Data analysis was performed using FlowJo flow cytometry analysis software (Tree Star, Ashland, OR). The viable cell population (population of interest) was determined by gating cells to exclude those with a low NADH auto fluorescence and those with high levels of SYTOX Green (cell death marker). The far-red median fluorescence intensity (MFI, Dylight 680 fluorophore) of the viable cell population was then assessed. Data points were collected in triplicate.

Cell surface isocyanoglycan labeling and quantification with fluorescence microscopy imaging

LL2 cells were seeded onto 4-well coverslip chambers (Lab-Tek™ borosylicate, Nunc, Roskilde, Denmark) at 2×10^{4}/cm^{2} and allowed to adhere to the plate surface for 6 hr. After adhesion, cells were pulsed with either isonitrile sugar or vehicle for 24 hr. Cells were then washed 3 times in ice cold FACS buffer before being incubated (30 min, 37 ^{o}C) *in situ* in 200 µL of FACS buffer containing 300 µM Tz-Biotin, followed by 2 washes as above and a subsequent incubation (15 min, 37 ^{o}C) with 50 µg/ml NeutrAvidin-Dylight680 and 300 nM DAPI (Invitrogen). After being washed as above, cells were fixed in PBS containing 4% formalin (RT, 15 min, 37 ^{o}C) and washed again twice in cold PBS. The

chambers were scanned on an iCys Research Imaging Cytometer (CompuCyte, Westwood, MA, U.S.A.) using 405 nm and 633 nm lasers. A 60x objective was used with 0.5 µm X-step size, giving a field size of 500 µm x 132 µm. A total of 300 fields were scanned for each chamber well. Watershed filters were also included in the protocol to ensure separate contouring on closely spaced cells. Images were analysed using primary and peripheral contours to sample cytoplasmic and membrane staining per cell respectively. The primary contour was set on the blue (DAPI) channel with an integration contour of 8 pixels to include cytoplasmic staining. The peripheral contour was set to a width of 8 pixels measuring from the outer edge of the integration contour. A sub contour was set with a threshold on the long red channel to measure total Alexa Fluor 647 staining per cell.

IncuCyte measurements of confluence

LL2 cells were seeded in a 96 well plate (Nunc, Roskilde, Denmark) at $1x10^4/cm^2$, 6 wells per sugar, in 100 µl of DMEM (as previously described) and incubated for 24 hrs in an IncuCyte HD (Essen Bioscience, Welwyn Garden City, UK). Isonitrile sugars were added in 100 µl of DMEM at 400 µM to give an overall sugar concentration of 200 µM. Scans were taken at 3 hour intervals, 4 scans per well.

S4

Flow cytometry data

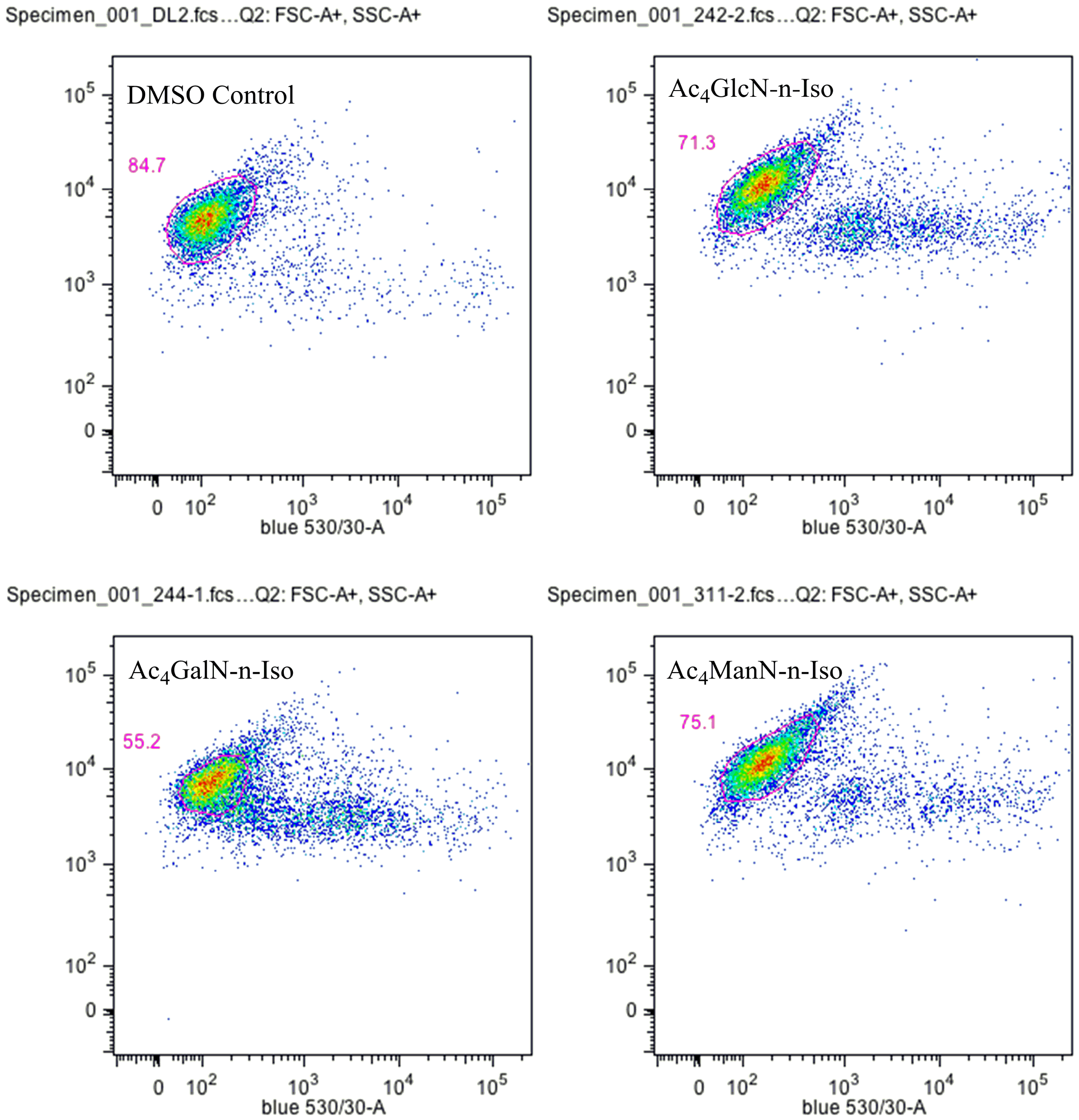

Figure S1. – Flow cytometry dot plots of labelling experiment described in Fig. 1. Each dot represents 1 of 10,000 events recorded. Y-axis measures NAD autofluorescence and X-axis measures Sytox Green, a cell death marker. Scales are logarithmic spanning 5 log units. Cells circled in pink were considered viable and are the only ones used to calculate the DyLight680 mean fluorescence intensity (MFI). The percentage of viable cells in the total population is shown in pink on each plot.

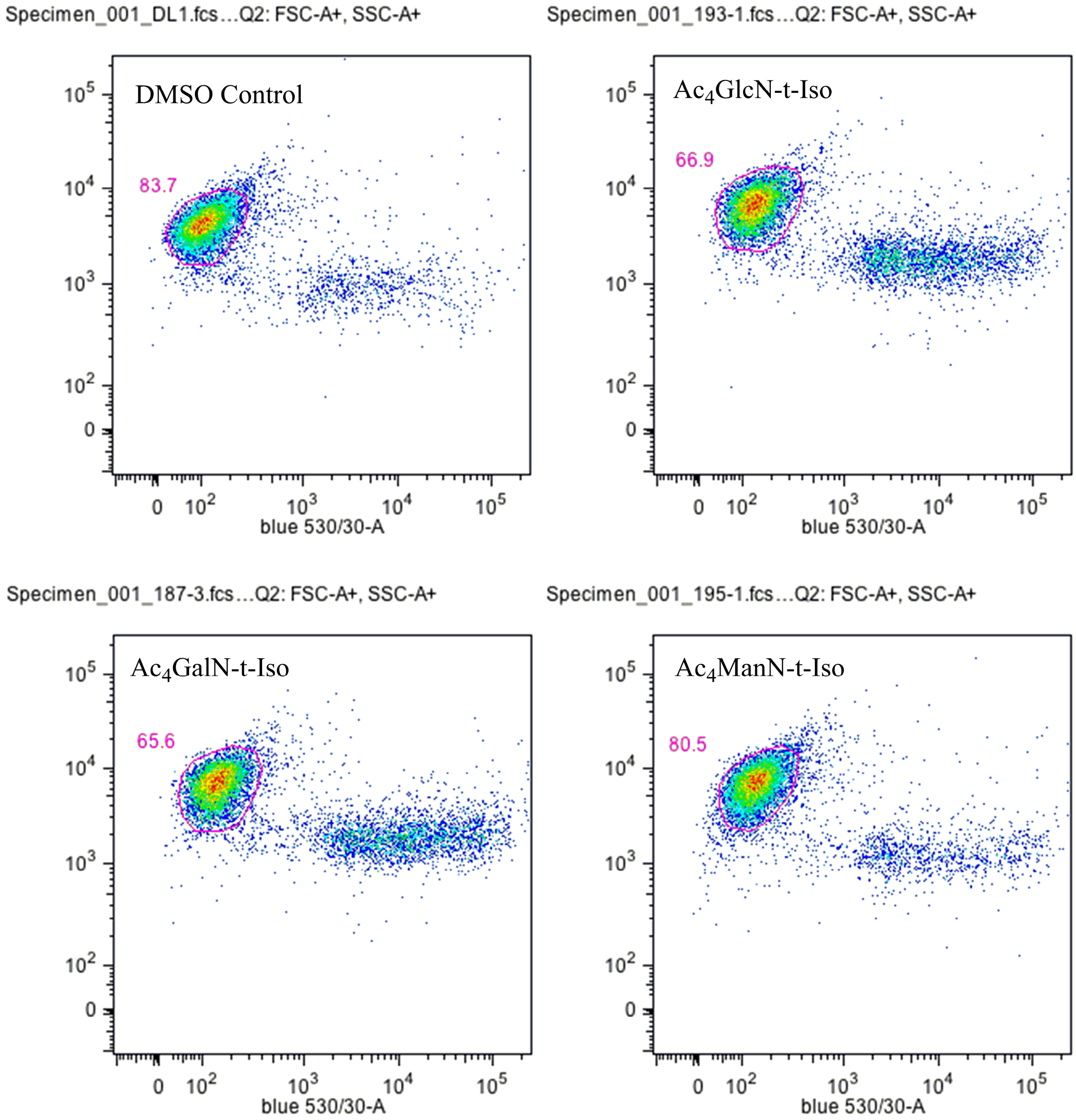

Figure S1. – continued

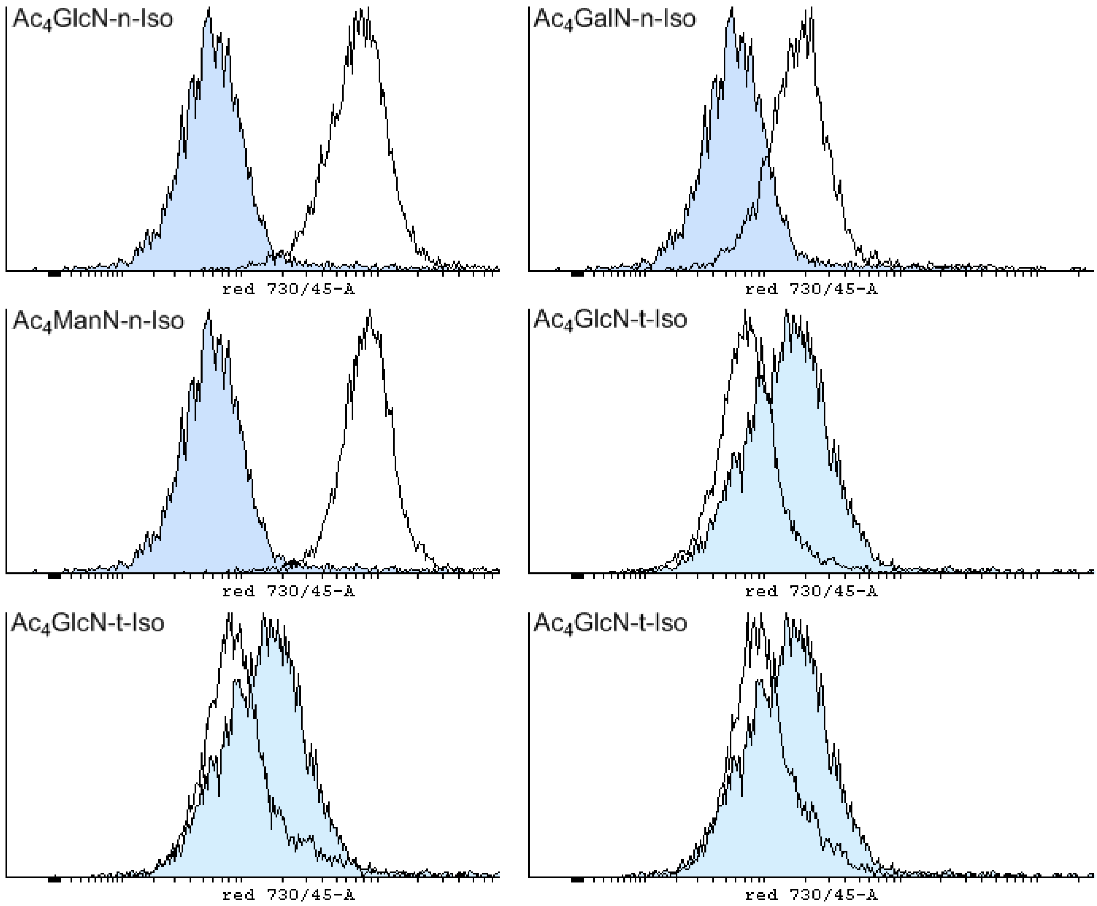

Figure S2 – Histograms from flow cytometry experiment described in Fig. 1. X-axis represents the absolute value recorded by laser 'red 730/45' which reports on NeutrAvidin-Dylight680; Y-axis is number of events. Blue histograms represent control cells which have been grown without added sugar whereas the unfilled histograms represent cells grown with the indicated sugar. Histograms represent only live cells gated for high NAD autofluorescence and low Sytox fluorescence as shown in fig. S1. Scale of X-axis is logarithmic spanning 5 log units. MFI's quoted in Fig. 1 are derived from the mean values of each histogram, averaged over 3 repeats.

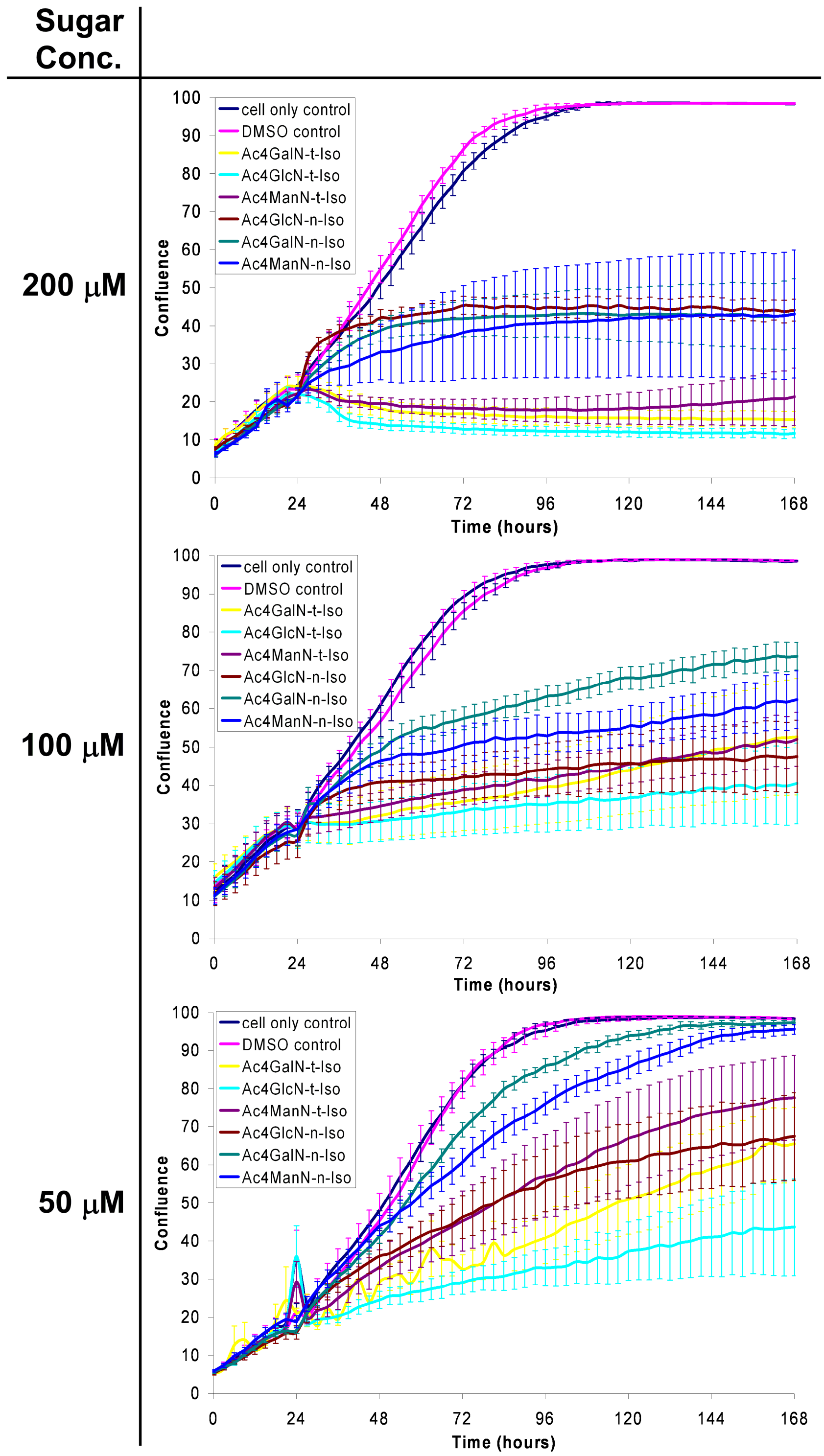

Figure S3 - Effect of the isonitrile sugars on cell growth. Cells were pulsed with concentration of sugar shown at t=24hr. Levels of confluence was measured at 3 h intervals. Error bars represent standard error across 6 repeats each of 4 scans.

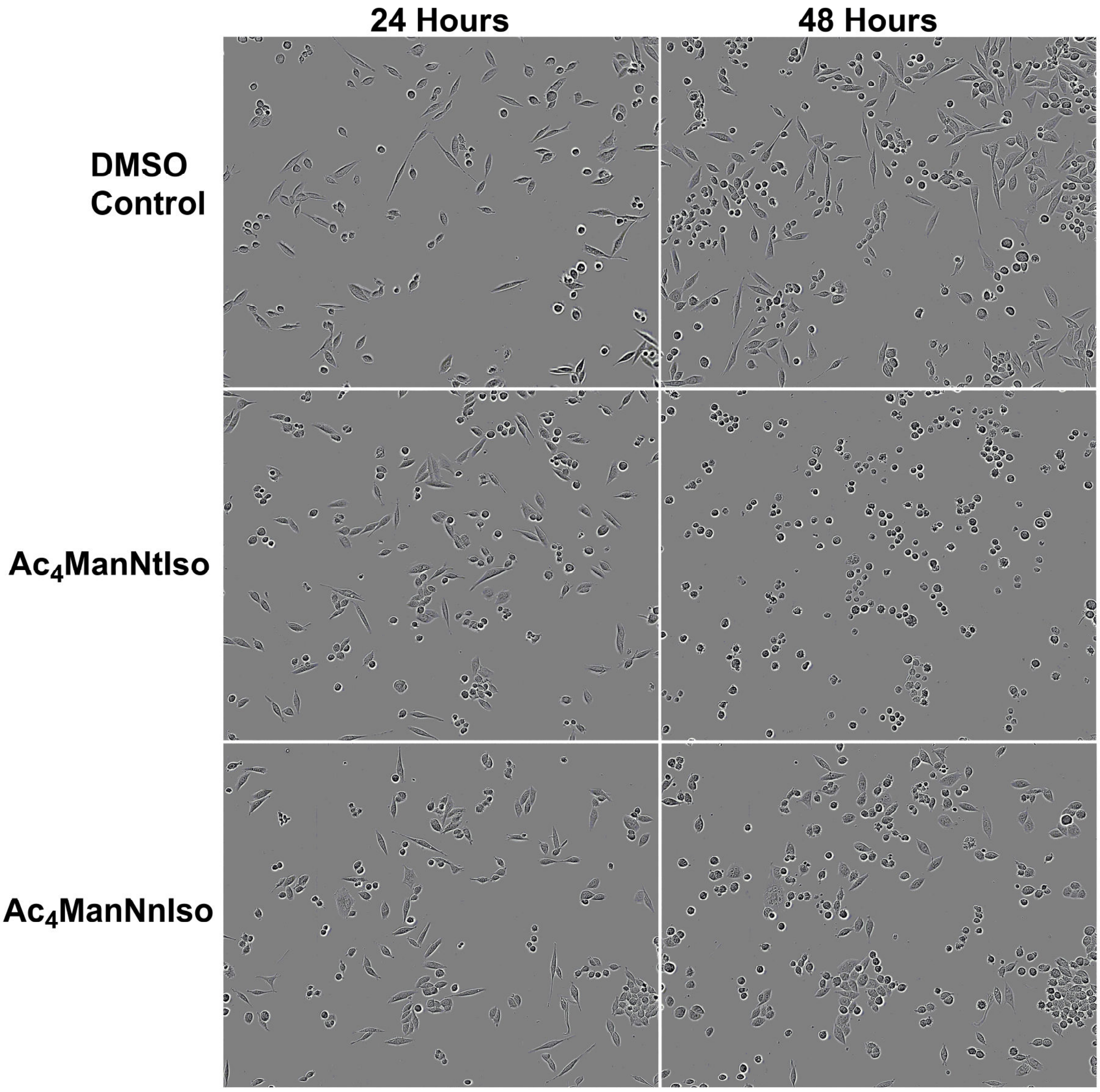

Figure S4 - Bright field microscopy images taken from the IncuCyte experiment described in Fig. 2.

	Live, gated cells as a percentage of the total number of cells					
	200 μM		100 μM		50 μM	
	Average	Error	Average	Error	Average	Error
Control	84.0	0.9	90.8	0.5	92.5	0.7
Ac_4GlcN-t-Iso	70.2	1.0	81.6	2.4	88.5	1.5
Ac_4GalN-t-Iso	58.6	2.6	82.3	1.0	88.1	0.5
Ac_4ManN-t-Iso	77.3	2.5	80.8	1.0	88.9	1.1
Control	85.8	0.7	92.2	2.0	92.1	0.6
Ac_4GlcN-n-Iso	64.1	5.8	89.2	1.4	85.5	0.3
Ac_4GalN-n-Iso	64.8	2.6	85.8	1.5	86.3	0.6
Ac_4ManN-n-Iso	82.4	1.4	88.3	3.4	88.3	0.3

Figure S5 - Percentages of viable cells selected during flow cytometry experiments described in Fig. 1.

S5

References

[1] B. Faroux-Corlay, L. Clary, C. Gadras, D. Hammache, J. Greiner, C. Santaella, A.-M. Aubertin, P. Vierling, J. Fantini, *Carbohydrate Res.*, **2000**, *327,* 223-260.
[2] T. Pirali, G. C. Tron, G. Masson, and J. Zhu, *Org. Lett.*, **2007**, *9*, 5275-5278.
[3] C. F. Hansell, I. A. Barker, A. P. Dove, R. K. O'Reilly, P. Espeel, M. M. Stamenovic, F. E. Du Prez, *J. Am. Chem. Soc.*, **2011**, *133*, 13828-13831.
[4] H. Stöckmann, A. A. Neves, H. A. Day, S. Stairs, K. M. Brindle, F. J. Leeper, *Chem. Comm.,* **2011**, *47*, 7203.
[5] R. Rossin, P. R. Verkerk, S. M. van den Bosch, R. C. M. Vulders, I. Verel, J. Lub, M. S. Robillard, *Angew. Chem. Int. Ed.,* **2010**, *49*, 3375.
[6] B. H. Rotstein, D. J. Winternheimer, A. K. Yudin, L. M. Yin, C. M. Deber, *Chem. Comm.*, **2012** , *48*, 3775-3777.
[7] J. O. Thomas, *Tetrahedron Lett.*, **1967**, *8*, 335-336.

S5

NMR Spectra

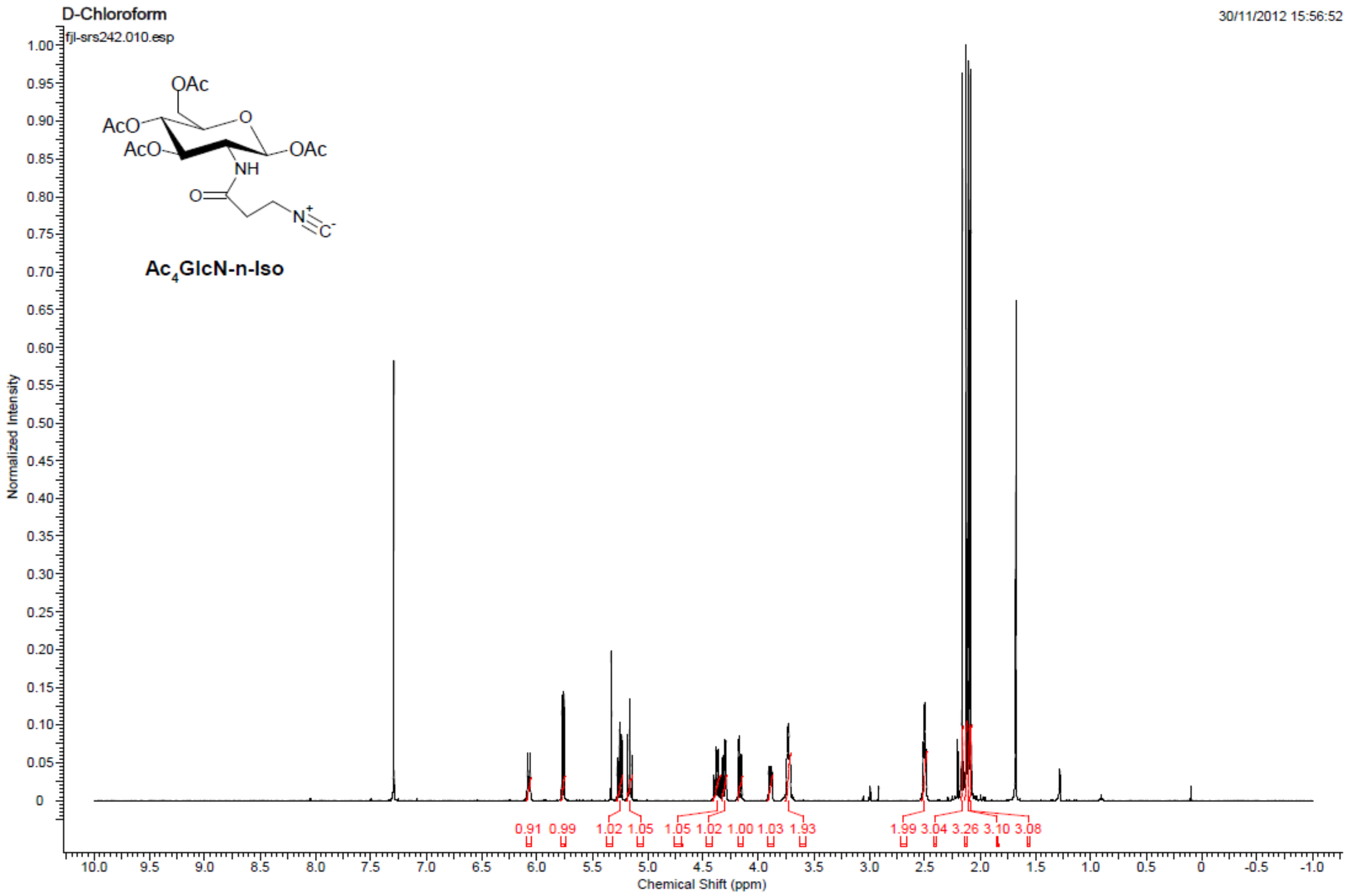
D-Chloroform
fjl-srs242.010.esp
30/11/2012 15:56:52
Ac4GlcN-n-Iso
Normalized Intensity
Chemical Shift (ppm)
0.91 0.99 1.02 1.05 1.05 1.02 1.00 1.03 1.93 1.99 3.04 3.26 3.10 3.08

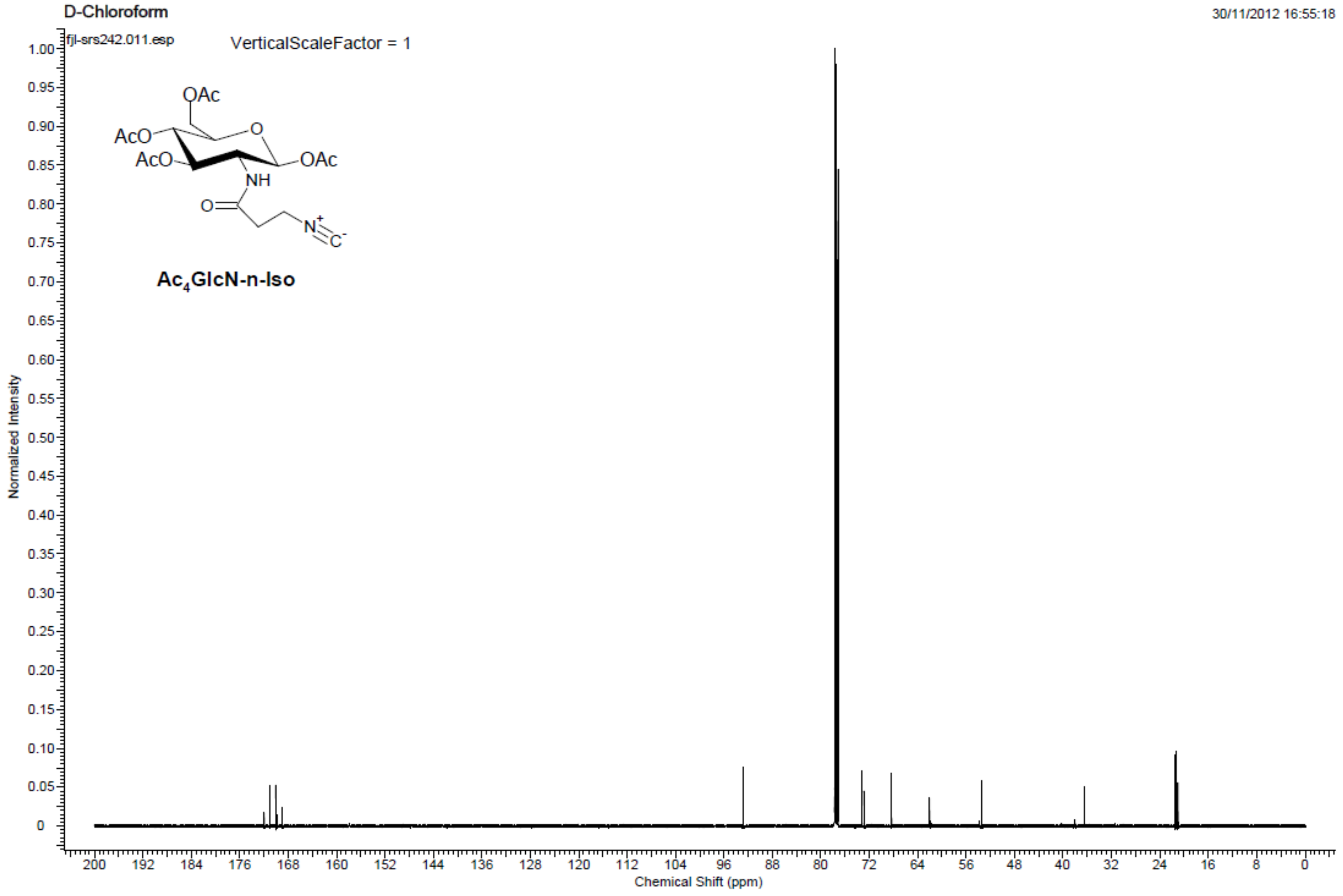
D-Chloroform
fjl-srs242.011.esp
VerticalScaleFactor = 1
30/11/2012 16:55:18
Ac4GlcN-n-Iso
Normalized Intensity
Chemical Shift (ppm)

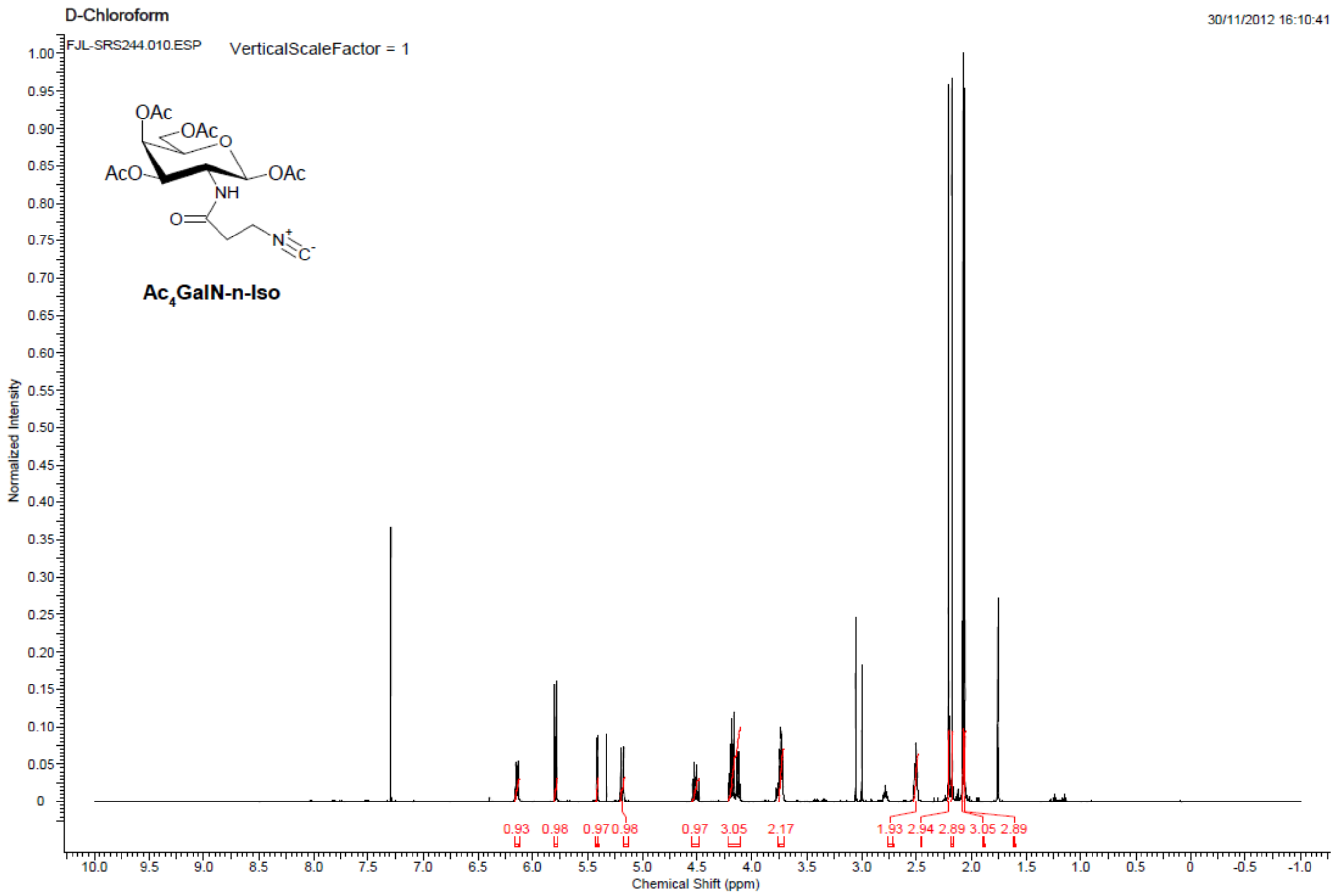
D-Chloroform
30/11/2012 16:10:41
FJL-SRS244.010.ESP
VerticalScaleFactor = 1
Ac$_4$GalN-n-Iso
0.93 0.98 0.97 0.98 0.97 3.05 2.17 1.93 2.94 2.89 3.05 2.89
Normalized Intensity
Chemical Shift (ppm)

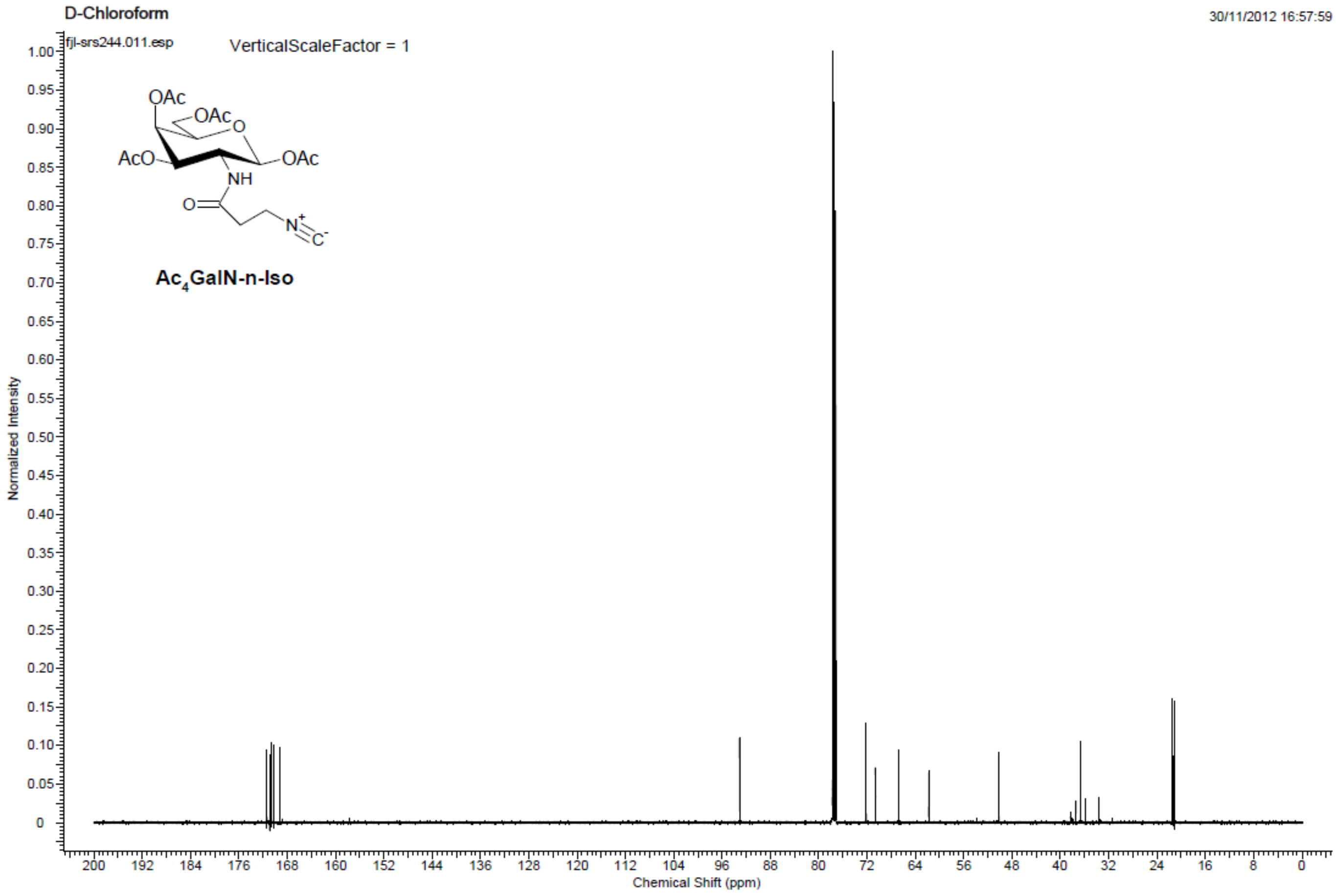
D-Chloroform
30/11/2012 16:57:59
fjl-srs244.011.esp
VerticalScaleFactor = 1
Ac$_4$GalN-n-Iso
Normalized Intensity
Chemical Shift (ppm)

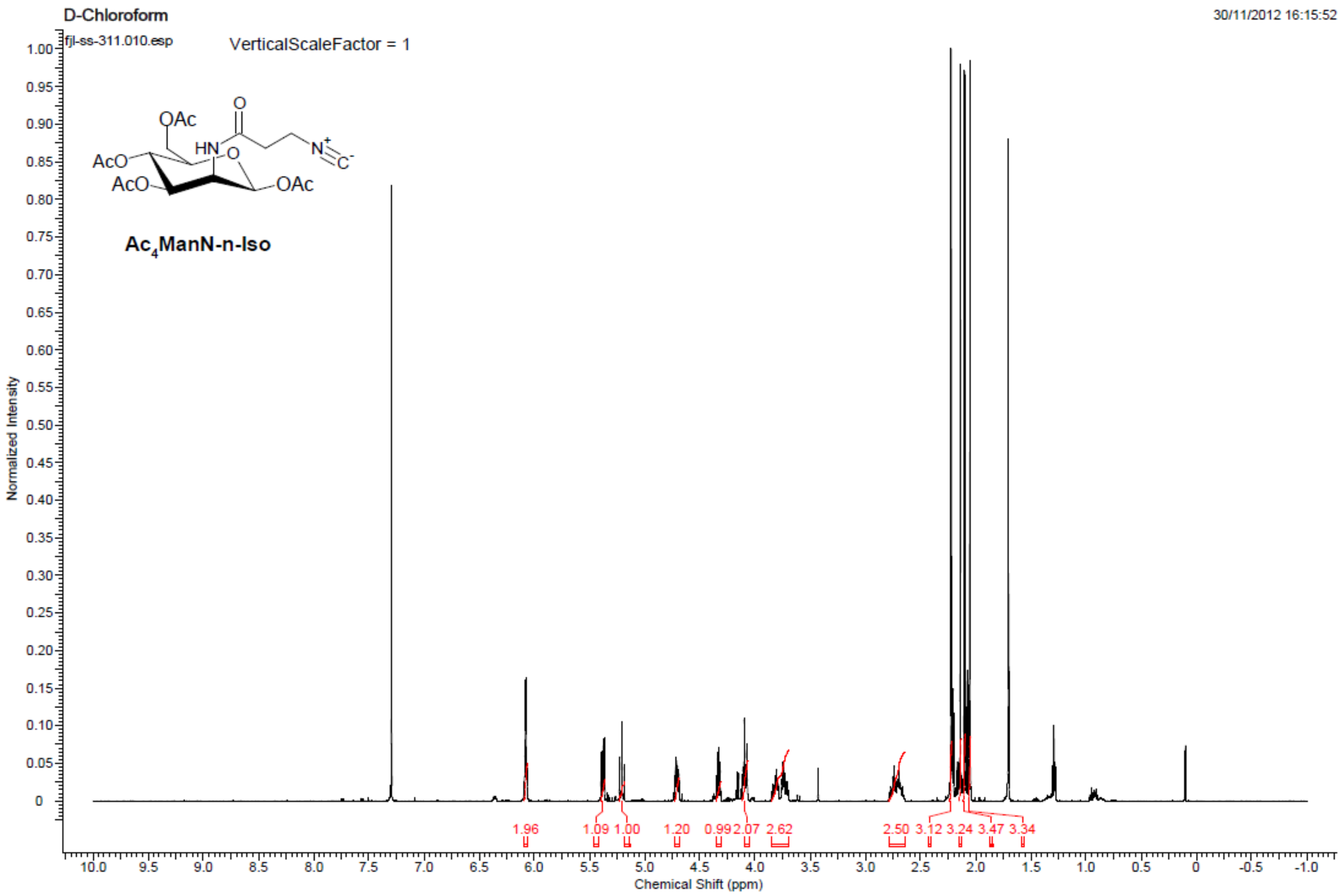
D-Chloroform
fjl-ss-311.010.esp
VerticalScaleFactor = 1
30/11/2012 16:15:52
Ac4ManN-n-Iso
OAc
AcO
AcO
HN
O
OAc
N
C
Normalized Intensity
Chemical Shift (ppm)
1.96 1.09 1.00 1.20 0.99 2.07 2.62 2.50 3.12 3.24 3.47 3.34

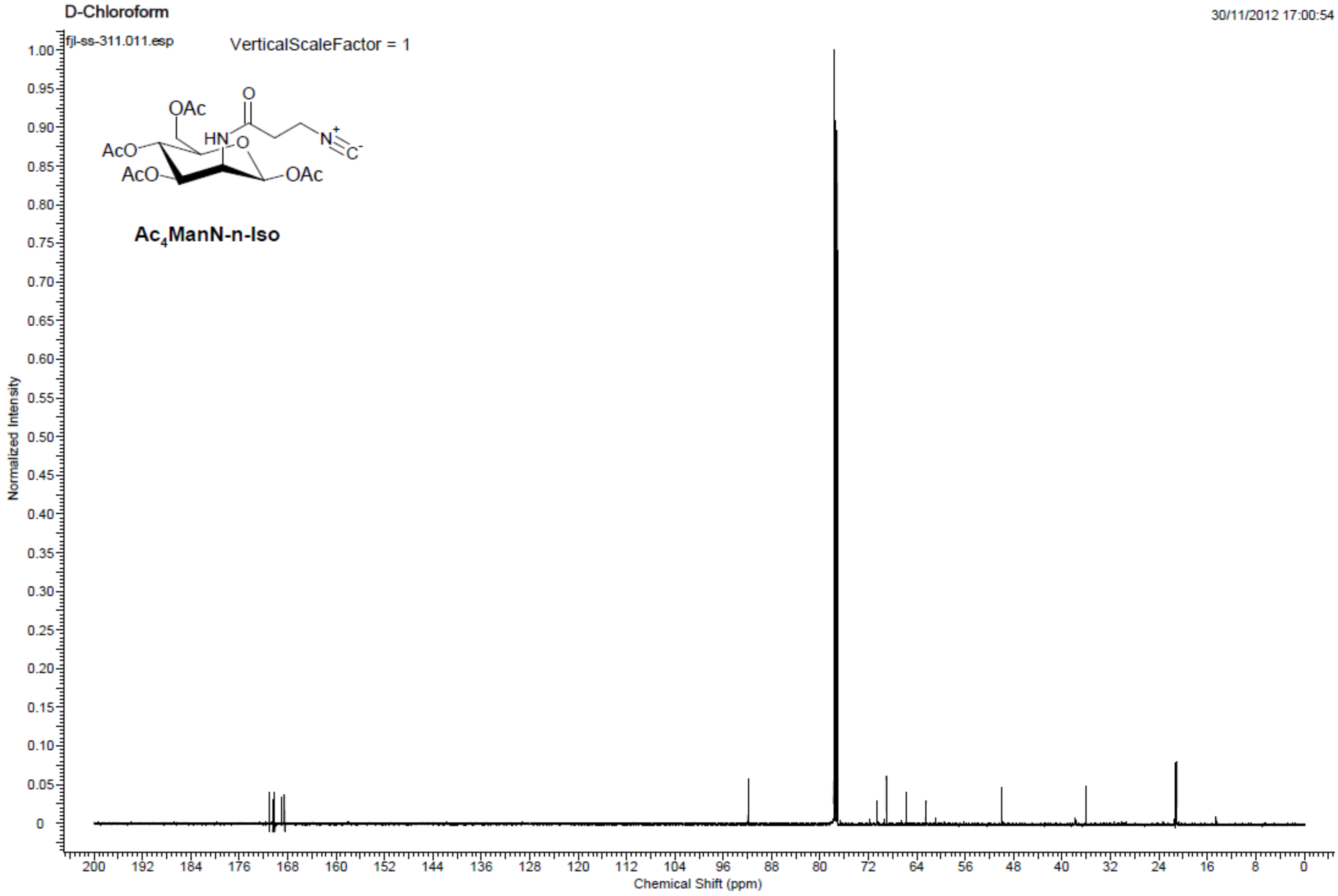
D-Chloroform
fjl-ss-311.011.esp
VerticalScaleFactor = 1
30/11/2012 17:00:54
Ac4ManN-n-Iso
OAc
AcO
AcO
HN
O
OAc
N
C
Normalized Intensity
Chemical Shift (ppm)

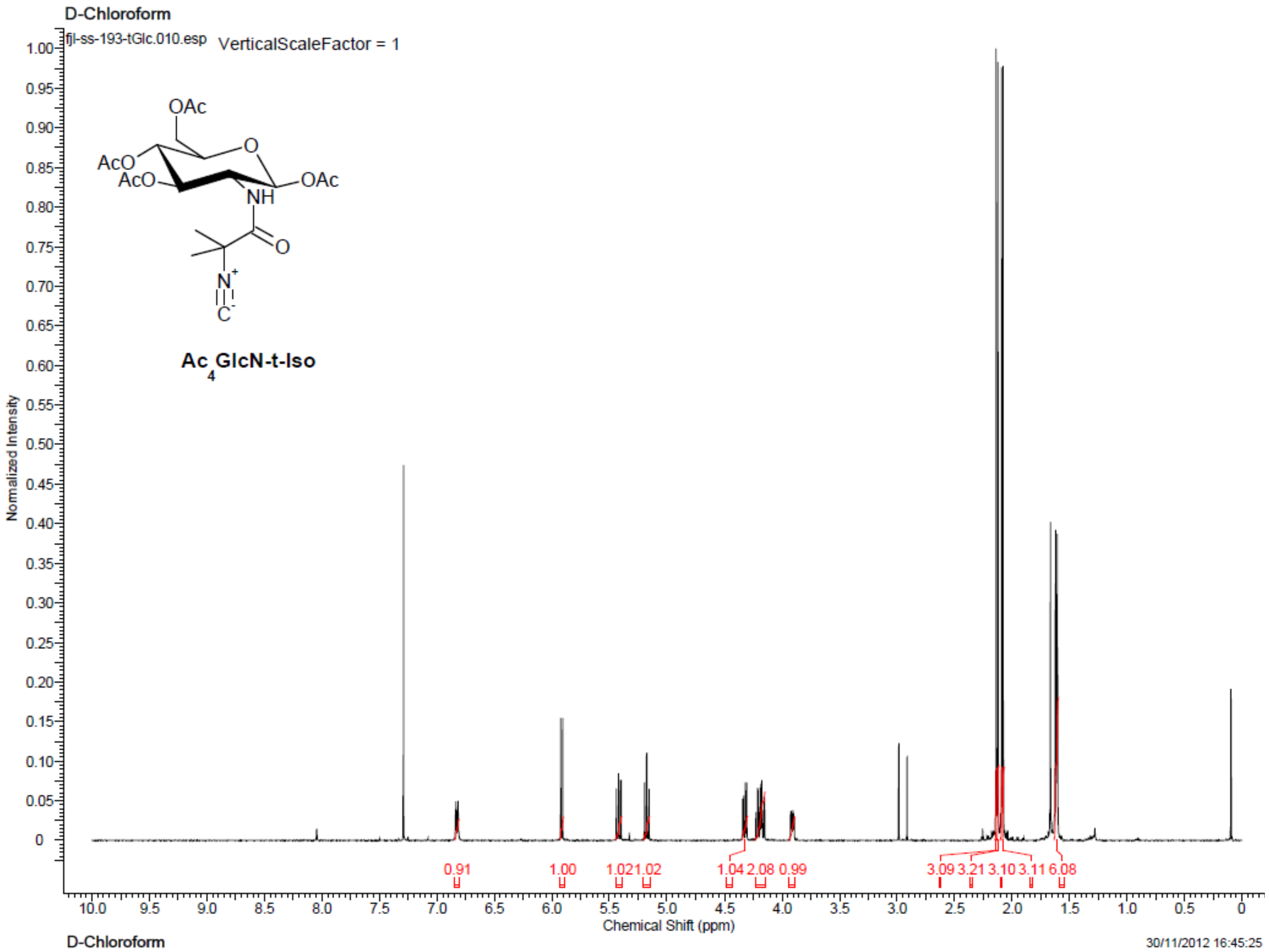
D-Chloroform
fjl-ss-193-tGlc.010.esp
VerticalScaleFactor = 1
OAc
AcO
AcO
O
OAc
NH
O
N+
C-
Ac4GlcN-t-Iso
Normalized Intensity
0.91
1.00
1.02
1.02
1.04
2.08
0.99
3.09
3.21
3.10
3.11
6.08
Chemical Shift (ppm)

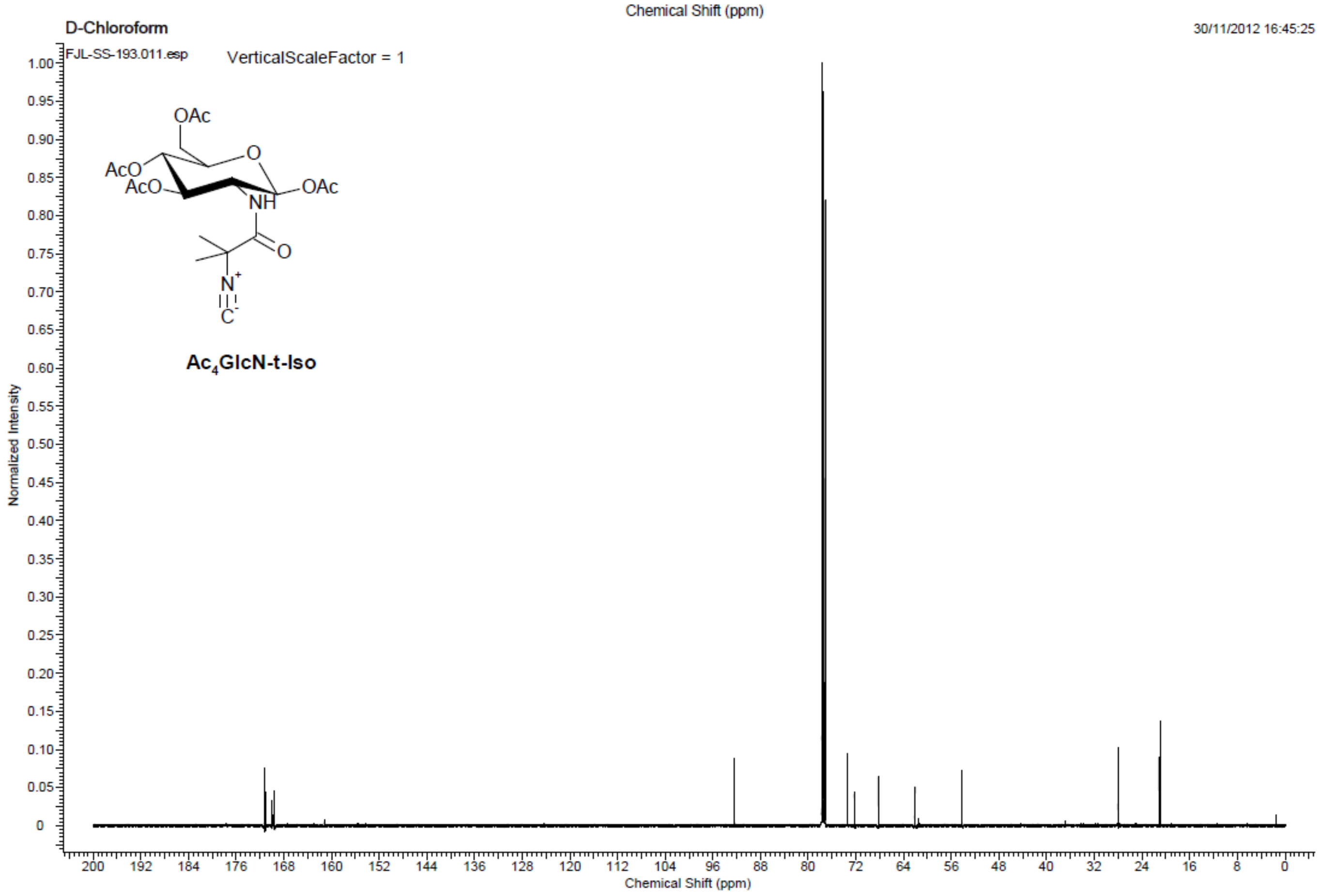
D-Chloroform
30/11/2012 16:45:25
FJL-SS-193.011.esp
VerticalScaleFactor = 1
OAc
AcO
AcO
O
OAc
NH
O
N+
C-
Ac4GlcN-t-Iso
Normalized Intensity
Chemical Shift (ppm)

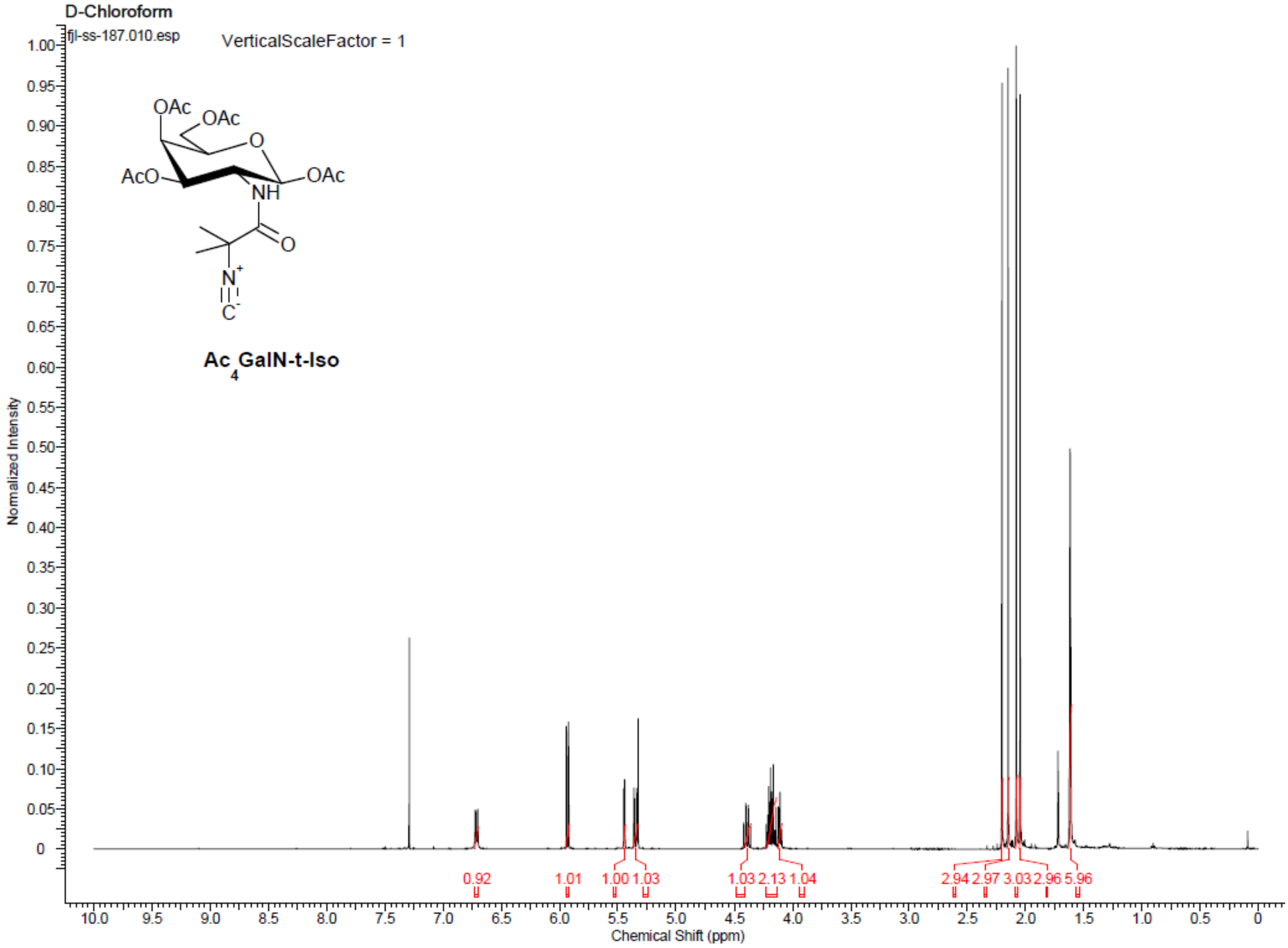
D-Chloroform
fjl-ss-187.010.esp
VerticalScaleFactor = 1
OAc
OAc
O
AcO
OAc
NH
O
N+
C-
Ac4GalN-t-Iso
Normalized Intensity
0.92 1.01 1.00 1.03 1.03 2.13 1.04 2.94 2.97 3.03 2.96 5.96
Chemical Shift (ppm)

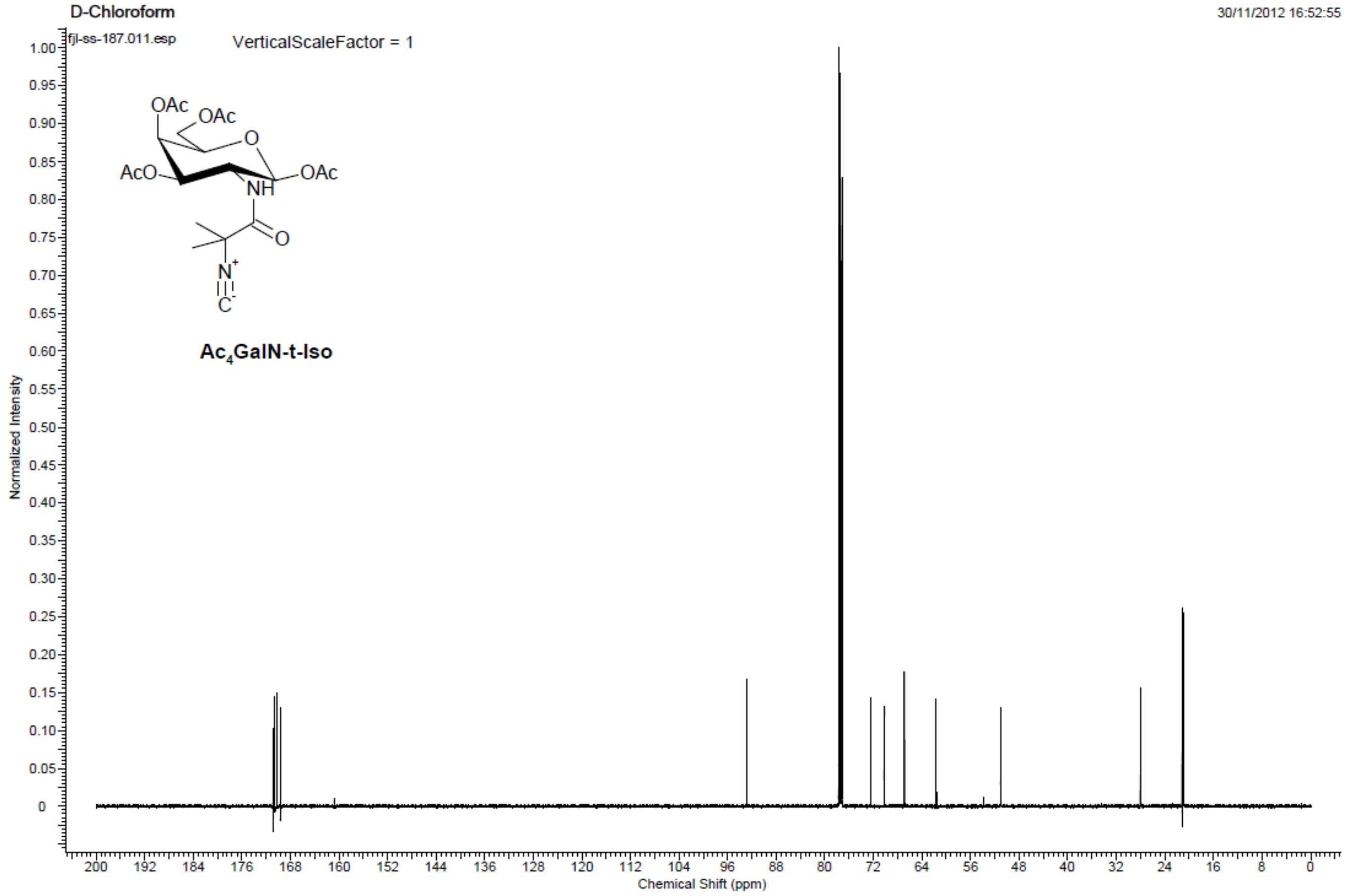
D-Chloroform
fjl-ss-187.011.esp
30/11/2012 16:52:55
VerticalScaleFactor = 1
OAc
OAc
O
AcO
OAc
NH
O
N+
C-
Ac4GalN-t-Iso
Normalized Intensity
Chemical Shift (ppm)

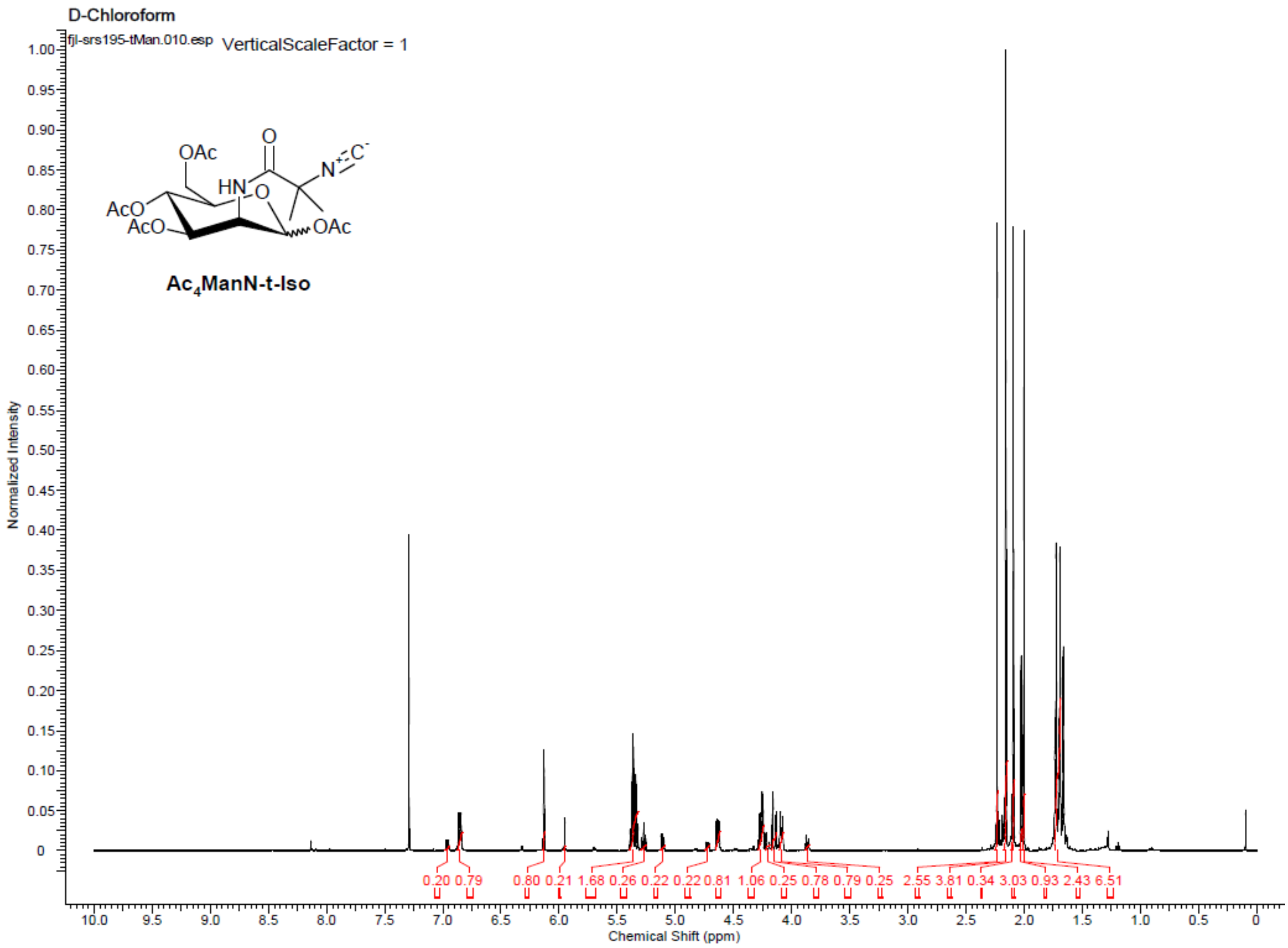
D-Chloroform
fjl-srs195-tMan.010.esp
VerticalScaleFactor = 1
OAc
O
N
C
HN
AcO
AcO
OAc
Ac4ManN-t-Iso
Normalized Intensity
0.20 0.79 0.80 0.21 1.68 0.26 0.22 0.22 0.81 1.06 0.25 0.78 0.79 0.25 2.55 3.81 0.34 3.03 0.93 2.43 6.51
Chemical Shift (ppm)

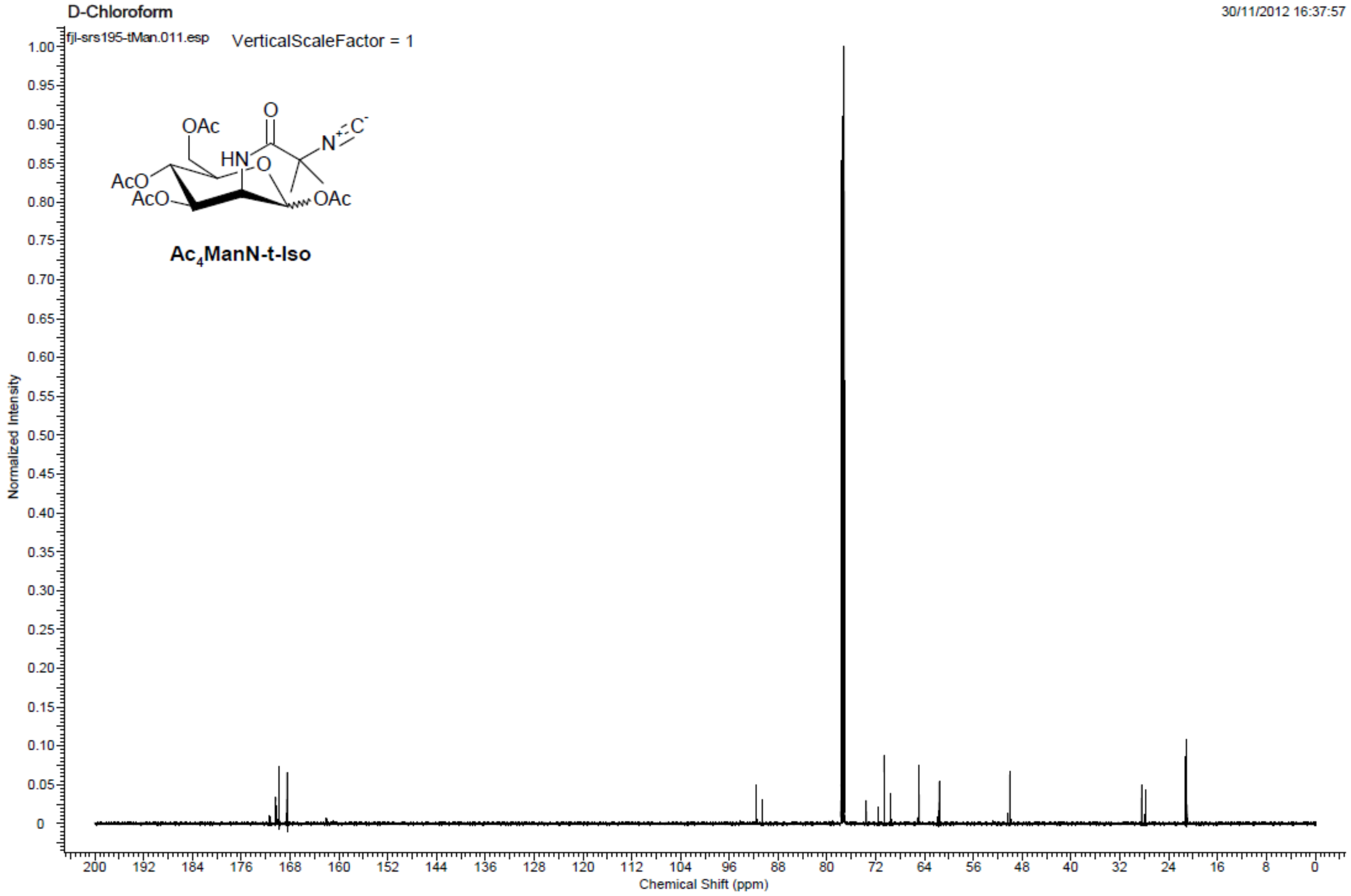
D-Chloroform
30/11/2012 16:37:57
fjl-srs195-tMan.011.esp
VerticalScaleFactor = 1
OAc
O
N
C
HN
AcO
AcO
OAc
Ac4ManN-t-Iso
Normalized Intensity
Chemical Shift (ppm)

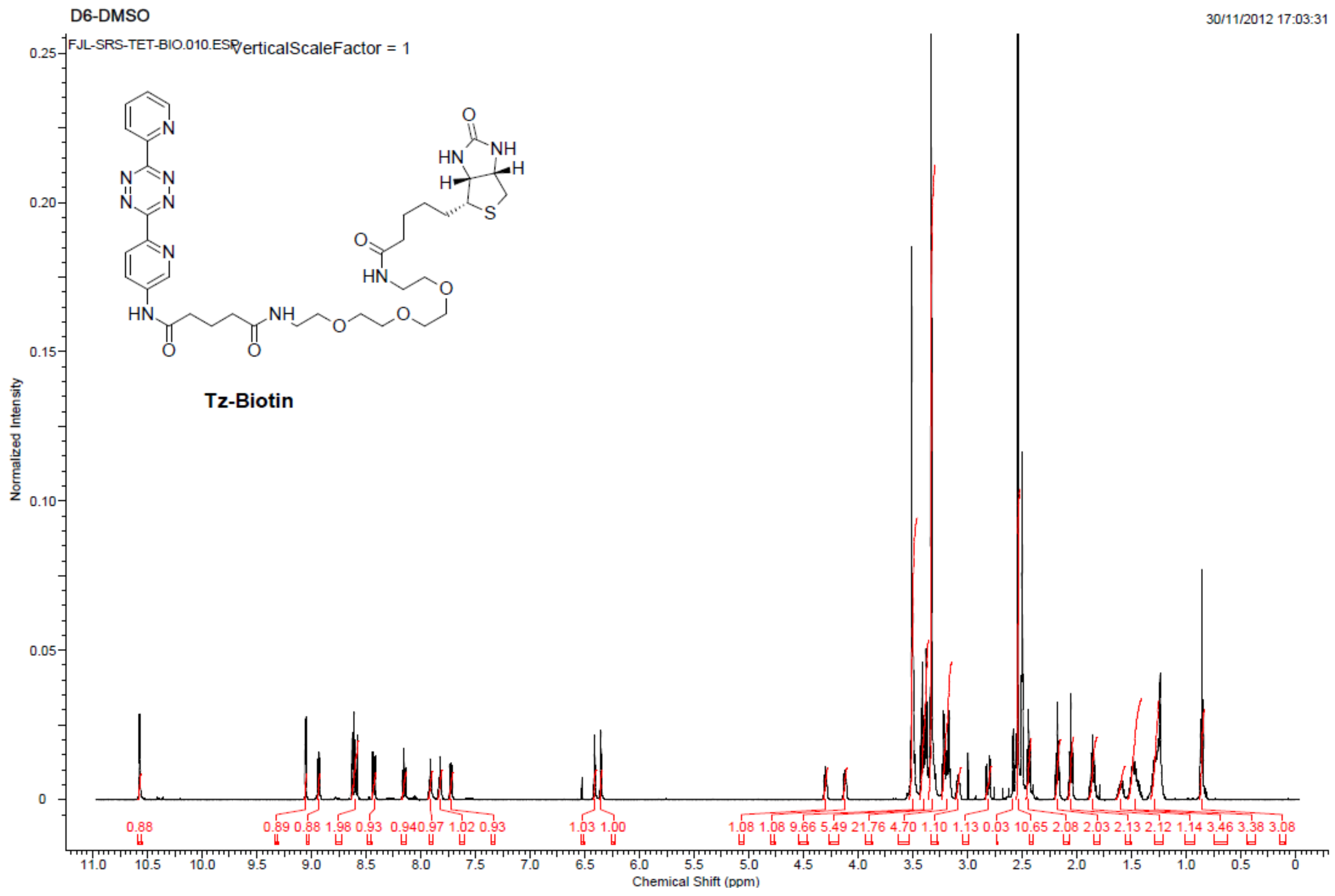
D6-DMSO
30/11/2012 17:03:31
FJL-SRS-TET-BIO.010.ESP
VerticalScaleFactor = 1
Tz-Biotin
Normalized Intensity
Chemical Shift (ppm)

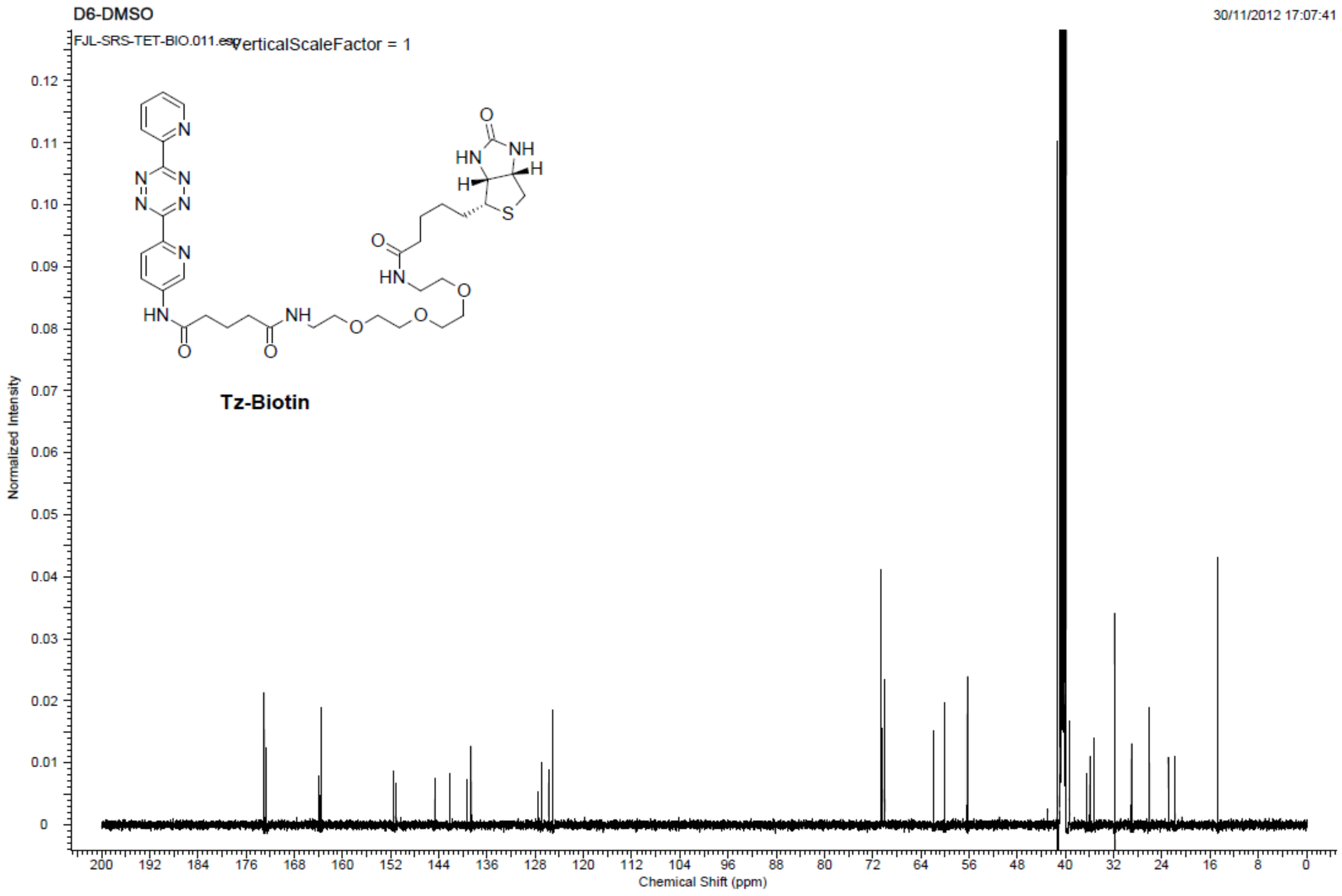
D6-DMSO
30/11/2012 17:07:41
FJL-SRS-TET-BIO.011.esp
VerticalScaleFactor = 1
Tz-Biotin
Normalized Intensity
Chemical Shift (ppm)

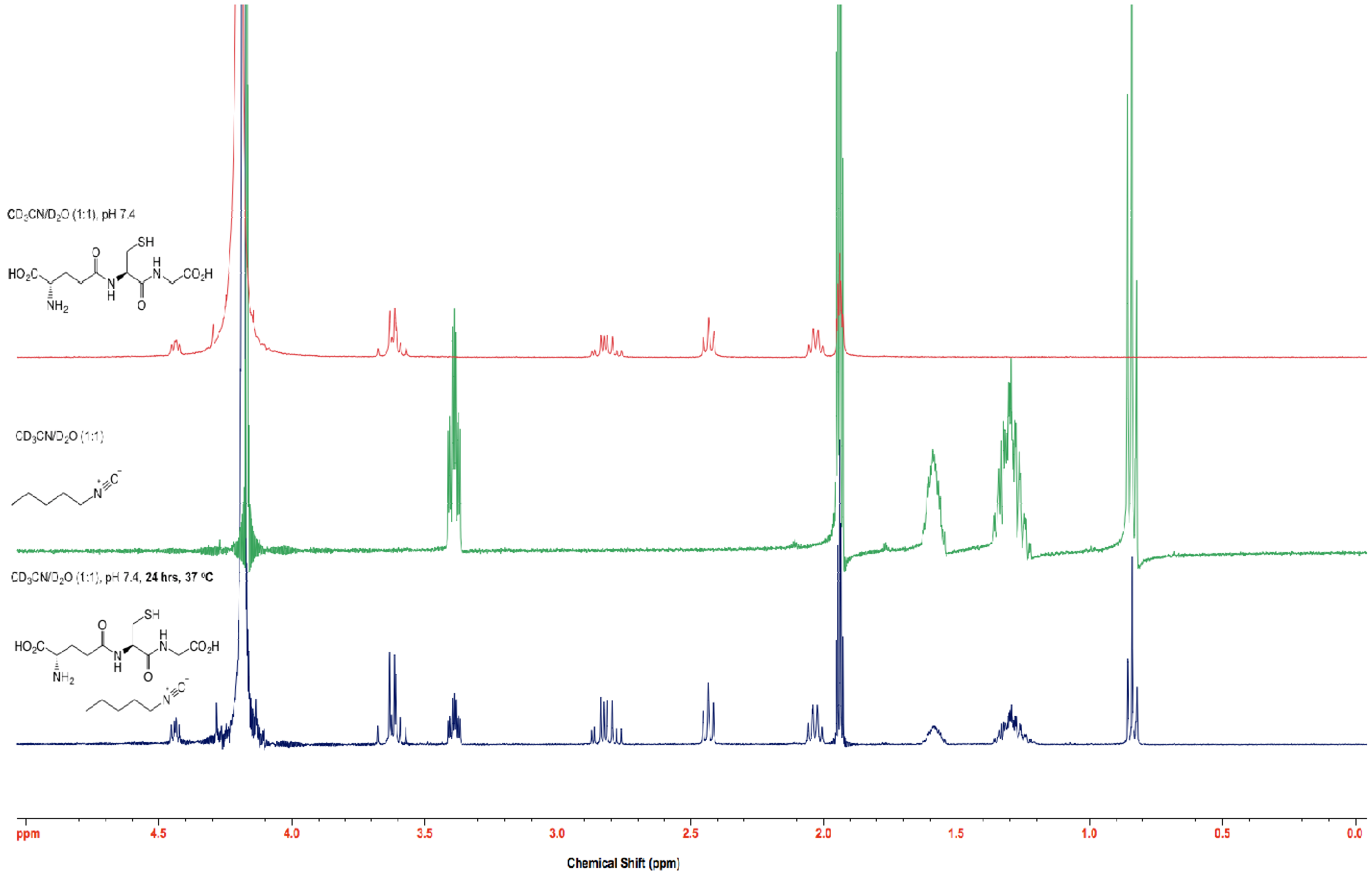
CD3CN/D2O (1:1), pH 7.4
CD3CN/D2O (1:1)
CD3CN/D2O (1:1), pH 7.4, 24 hrs, 37 °C
ppm
4.5
4.0
3.5
3.0
2.5
2.0
1.5
1.0
0.5
0.0
Chemical Shift (ppm)

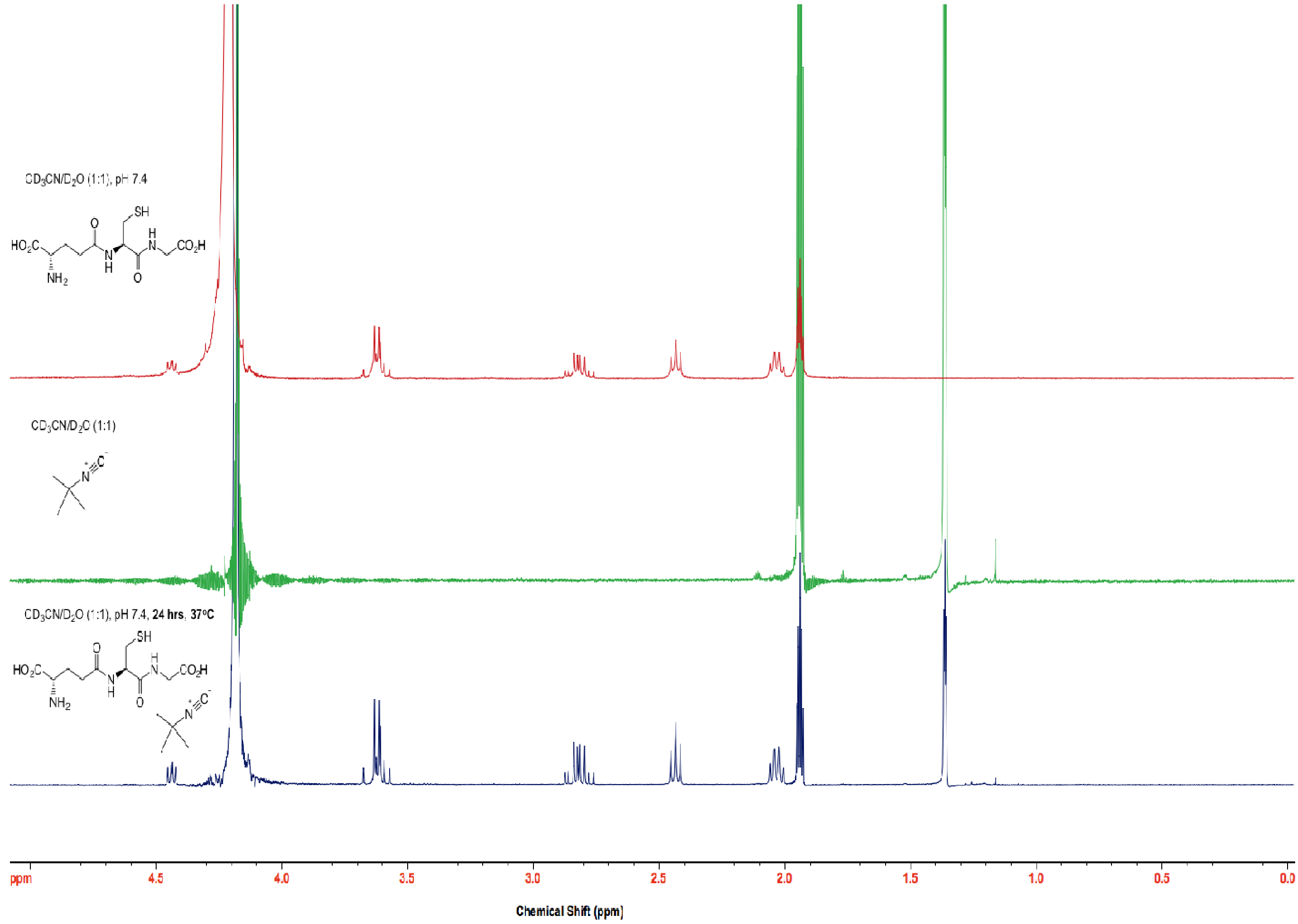
CD3CN/D2O (1:1), pH 7.4
CD3CN/D2O (1:1)
CD3CN/D2O (1:1), pH 7.4, 24 hrs, 37°C
ppm
4.5
4.0
3.5
3.0
2.5
2.0
1.5
1.0
0.5
0.0
Chemical Shift (ppm)

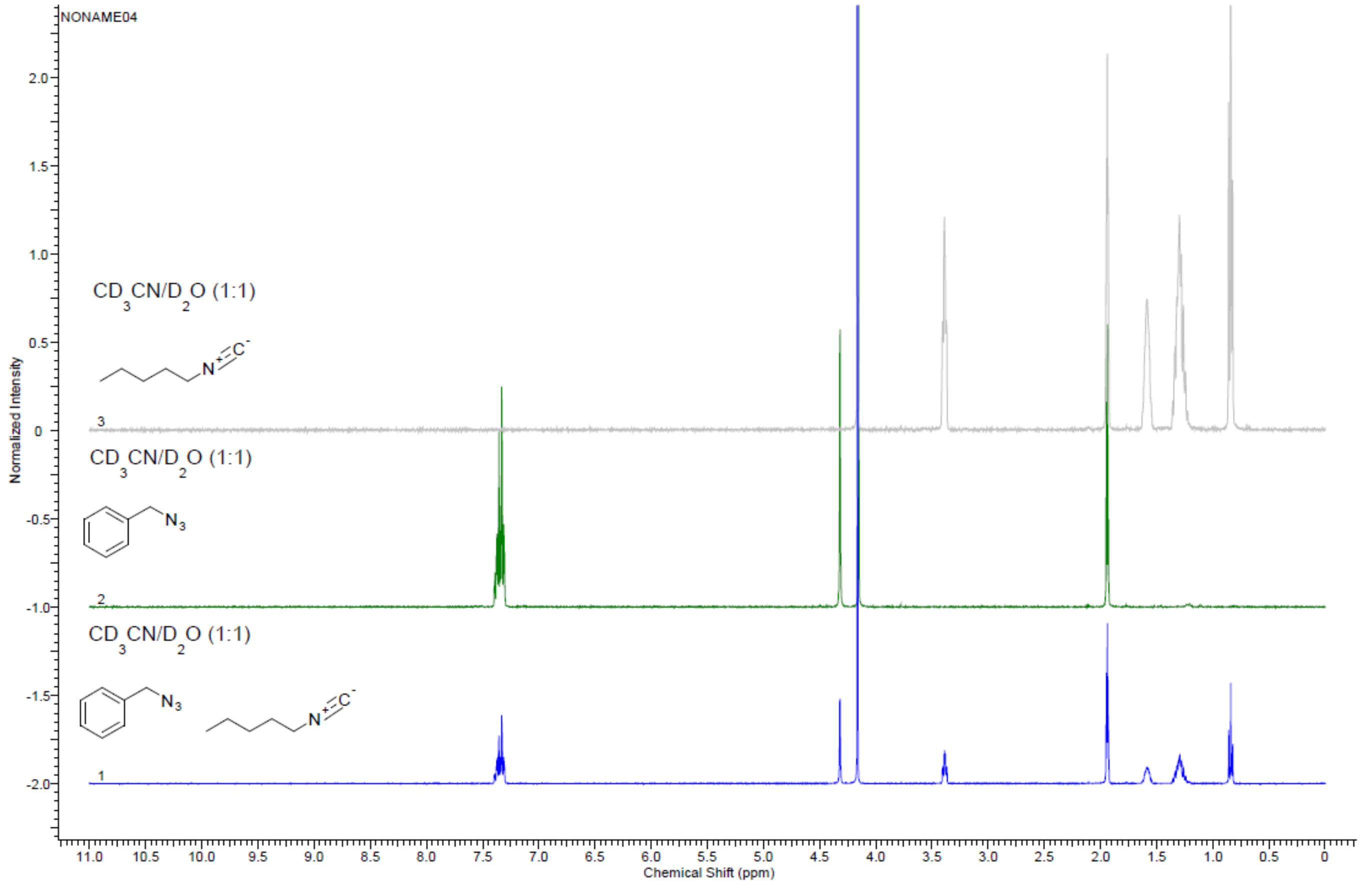
NONAME04
Normalized Intensity
CD3CN/D2O (1:1)
CD3CN/D2O (1:1)
CD3CN/D2O (1:1)
N3
Chemical Shift (ppm)

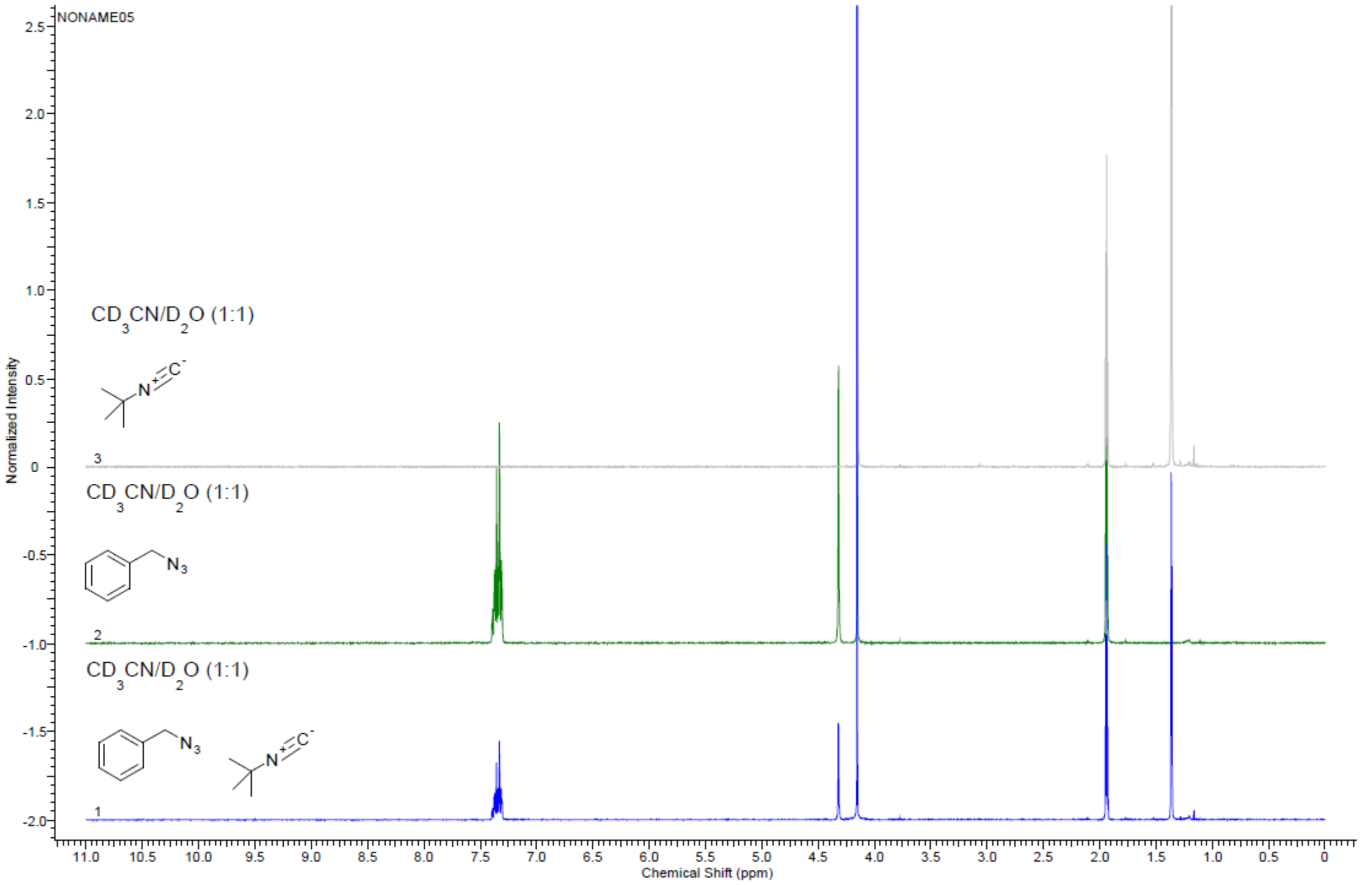
NONAME05
Normalized Intensity
CD3CN/D2O (1:1)
CD3CN/D2O (1:1)
CD3CN/D2O (1:1)
N3
Chemical Shift (ppm)

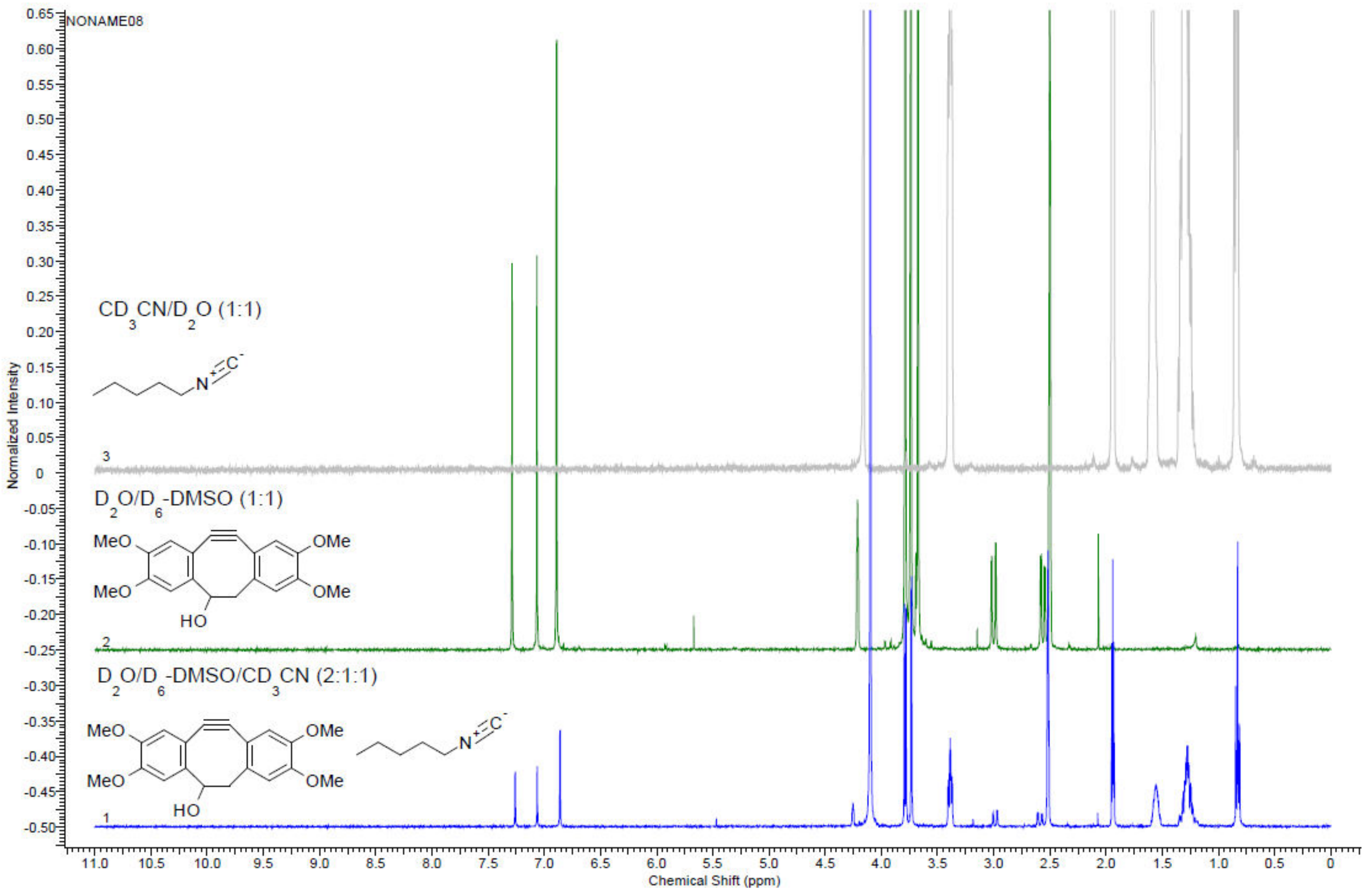
NONAME08
CD3CN/D2O (1:1)
D2O/D6-DMSO (1:1)
D2O/D6-DMSO/CD3CN (2:1:1)
MeO
OMe
HO
Normalized Intensity
Chemical Shift (ppm)

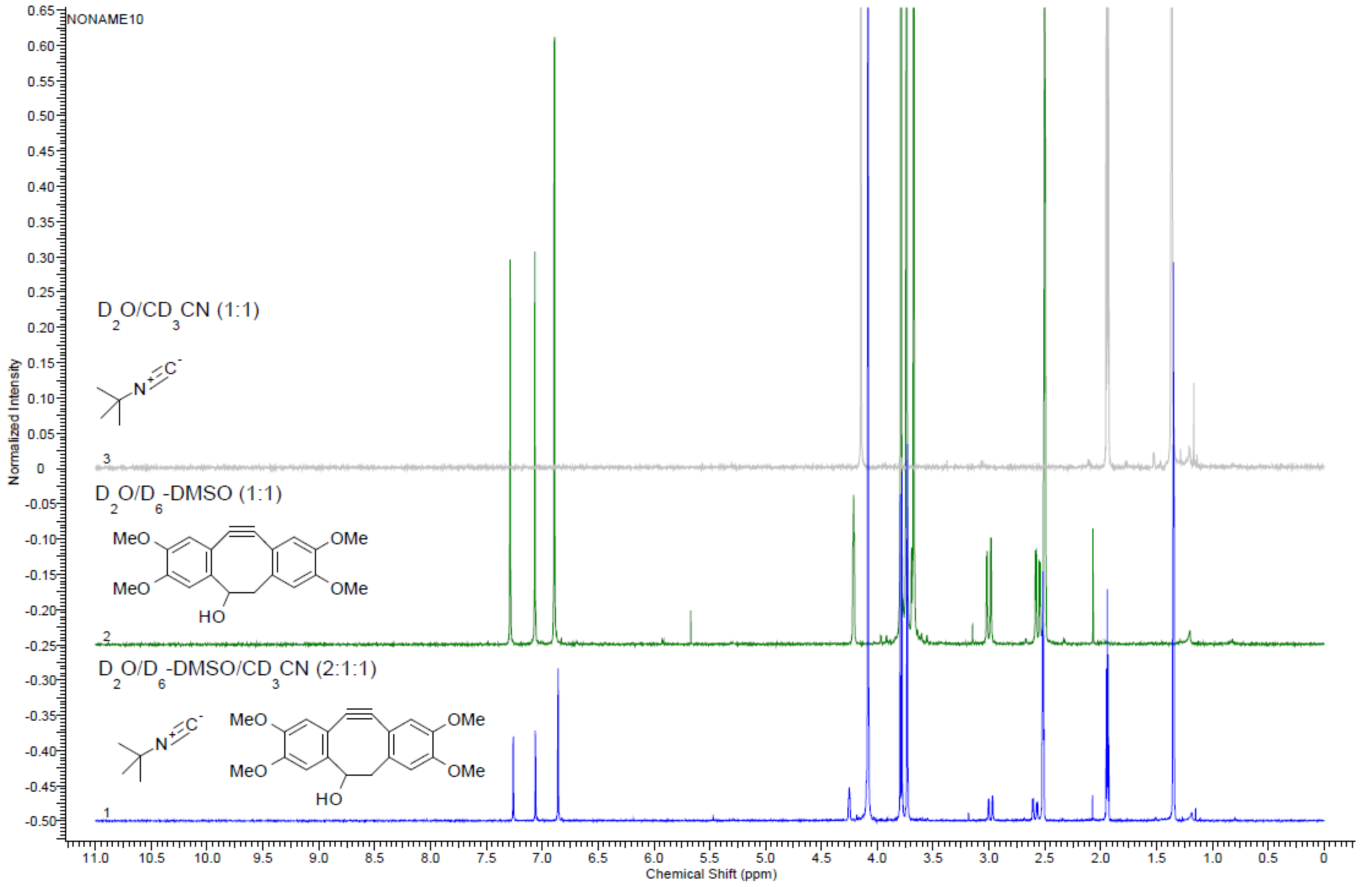
NONAME10
D2O/CD3CN (1:1)
D2O/D6-DMSO (1:1)
D2O/D6-DMSO/CD3CN (2:1:1)
MeO
OMe
HO
Normalized Intensity
Chemical Shift (ppm)
